# Anthropometric, Body Composition, and Nutritional Indicators with and without Nutritional Intervention during Nitisinone Therapy in Alkaptonuria

**DOI:** 10.3390/nu16162722

**Published:** 2024-08-15

**Authors:** L. R. Ranganath, M. Khedr, A. M. Milan, A. S. Davison, A. T. Hughes, B. P. Norman, H. Bygott, E. Luangrath, S. Judd, C. Soulsby, B. Olsson, R. Imrich

**Affiliations:** 1 Department of Clinical Biochemistry and Metabolic Medicine, Royal Liverpool University Hospital, Prescot Street, Liverpool L7 8YE, UK; 2 Department of Musculoskeletal and Ageing Science, Institute of Life Course and Medical Sciences, University of Liverpool, Liverpool L7 8TX, UK; 3 Department of Nutrition & Dietetics, Royal Liverpool University Hospital, Prescot Street, Liverpool L7 8YE, UK; 4 Garriguella AB, 179 62 Ekerö, Sweden; 5 Institute of Clinical and Translational Research, Biomedical Research Center, Slovak Academy of Sciences, 84104 Bratislava, Slovakia; 6 Faculty of Medicine of Comenius University, 81372 Bratislava, Slovakia

**Keywords:** alkaptonuria, nitisinone, tyrosinaemia, nutrition, body composition

## Abstract

Introduction: Protein nutrition disorder in alkaptonuria (AKU), resulting in increased homogentisic acid (HGA) before nitisinone therapy and increased tyrosine (TYR) during nitisinone therapy, may benefit from dietetic intervention. The aim of this study was to characterise the diet and their effects prospectively in those who received formal dietetic intervention in the nitisinone-receiving National Alkaptonuria Centre (NAC) patients with those who did not in no-nitisinone Suitability of Nitisinone in Alkaptonuria 2 (SN2 N−) and nitisinone-treated SN2 (SN2 N+) randomised study groups. Patients and methods: A total of 63, 69, and 69 AKU patients from the NAC, SN2 N−, and SN2 N+ were studied for anthropometric (weight, BMI), body composition (including muscle mass, %body fat, hand grip strength), chemical characteristics (serum TYR, serum phenylalanine, urine urea or uUREA, and urine creatinine or uCREAT), and corneal keratopathy. Nitisinone 2 mg and 10 mg were employed in the NAC and SN2 N+ groups, respectively. Dieticians managed protein intake in the NAC, while the SN2 N− and SN2 N+ groups only received advice on self-directed protein restriction during four years of study duration. Results: uUREA decreased in the NAC, SN2 N−, and SN2 N+ groups, showing that protein restriction was achieved in these groups. Body weight and BMI increased in the NAC and SN2 N+ groups. uCREAT decreased significantly in SN2 N− and SN2 N+ compared with the NAC over four years of study. Corneal keratopathy was less frequent in the NAC than in the SN2 N+ group. Active dietetic intervention in NAC stabilised lean body mass (muscle mass, hand grip strength) despite a decrease in uUREA and uCREAT, as well as sTYR. Conclusion: Ongoing dietetic intervention prevented loss of lean body mass despite protein restriction and moderated serum tyrosine increase, leading to less prevalent corneal keratopathy. Protein restriction risks fat mass gain.

## 1. Introduction

Alkaptonuria (AKU) is an autosomal recessive disorder affecting the phenylalanine/tyrosine (PHE/TYR) catabolic pathway with a worldwide prevalence of around 1 in 250,000 [1]. A deficiency of homogentisate dioxygenase (HGD) activity leads to an inability to catabolise homogentisic acid (HGA) (Figure S1) [2]. Manifestations of AKU are due to HGA itself or to the conversion of HGA to a melanin-like pigment in a process called ochronosis [3,4]. The resulting accumulation of HGA causes the multisystem morbidity characterised by stones (kidney, prostate, salivary, gall bladder), aortic stenosis, osteopenia, fractures, ruptures (tendons, ligaments and muscle), and spondyloarthropathy [5].

Therefore, lowering HGA is a key therapeutic strategy. Before effective HGA-lowering options were available, low protein diets were a mainstay in the management of AKU, despite being unproven in terms of modifying AKU disease outcomes [6]. Nevertheless, such ineffective low protein strategies are still the norm and result in malnutrition in AKU [7].

A recently approved therapy for AKU using a drug called nitisinone, an inhibitor of enzyme 4-hyroxyphenylpyruvate dioxygenase (HPPD) (Figure S1), effectively lowers circulating and urinary HGA depending upon the dose used by 95–99% [8,9]. Further, HGA-lowering also reduces the disease process ochronosis and crucially improves patient outcomes by slowing disease progression [9]. However, HPPD inhibition blocks the PHE/TYR pathway proximal to HGA production, thereby increasing 4-hyroxyphenylpyruvate and tyrosine [10]. Nitisinone-induced tyrosinaemia has been associated with the well-known reversible dendritiform corneal keratopathy associated with painful red eye syndrome [9,11]. To counter the nitisinone-induced tyrosinaemia, dietary restriction of PHE and TYR, as well as both protein restriction and PHE/TYR-free protein substitutes, are employed.

All patients with AKU in the United Kingdom are eligible to receive nitisinone in the National Alkaptonuria Centre (NAC) and require dietetic management. Dietetic management involves a controlled protein intake of 0.75 to 1.0 g protein per kg body weight per day. Recommended protein intake is based on the UK reference nutrient intake and the World Health Organization recommended daily allowance and is the minimum safe protein intake. PHE/TYR-free protein substitutes are not used as standard but are usually added to help manage high serum tyrosine levels and are used in approximately one-third of the patients (Table S1) [12]. There is, therefore, a lack of a no-nitisinone control group to compare nutritional management of tyrosinaemia in the NAC. A recent publication reported on 69 patients in the nitisinone-treated group in the Suitability of Nitisinone in Alkaptonuria 2 (SONIA 2 or SN2) study showing decreased daily urinary creatinine (uCREAT) (an indicator of muscle mass), as well as decreased daily urinary urea (uUREA) (an indicator of protein intake) [13]. The diet was not actively managed in SN2, unlike the NAC, where an experienced dietician has been in place since the beginning. Unlike the NAC, an additional 69 patients in the SN2 study, so far not reported in full, were untreated and followed up for four years, where body weight, BMI, uUREA, and uCREAT were measured annually for four years. Body composition data and other nutritional indicators were measured only in the NAC and not in the SN2.

The aim of the present analysis was to characterise the dietary management in the NAC nitisinone group with the untreated and nitisinone-treated groups of the SN2 study over four years. The efficacy in lowering serum tyrosine to acceptable levels and safety in terms of minimising sarcopenia and maintaining muscle function was assessed during nutritional management of nitisinone therapy in the NAC.

## 2. Methods

### 2.1. The SN2 Study Cohort

SN2 was a four-year, open-label, evaluator-blinded, multicentre, randomised, no-treatment controlled, parallel-group study [9]. The study sites were Liverpool (UK), Paris (France), and Piešťany (Slovakia). This study included 138 patients, 69 in control (SN2 N−) and 69 randomised to oral nitisinone (SN2 N+) 10 mg daily, aged 25 years or older, with diagnosed AKU, and any clinical manifestation in addition to increased HGA. The visits were once a year (baseline and months 12, 24, 36, and 48). Independent ethics committees at each study site approved this study. All patients provided written informed consent prior to inclusion.

### 2.2. The NAC Cohort

Sixty-three patients with biochemically confirmed AKU, namely a documented increase in urine homogentisic acid, aged 16 years and over, attended the RLUH between 2007 and 2023 for at least four years. The NAC provides oral nitisinone, off-licence, mainly 2 mg daily, but some have been on 5 mg and 10 mg doses since 2022. No patient received nitisinone prior to 2012. All patients were on nitisinone. The duration of nitisinone 2 mg therapy varied among the patients. Only those NAC patients who had completed four years of nitisnone therapy, matching the duration in SN2, were included for analysis in the current dataset.

### 2.3. Assessment at Every Visit

Demographic, body weight, and BMI data were available for both the NAC and SN2 cohorts. In both the NAC and SN2, measurements of uUREA and uCREAT were assayed using Roche Cobas analysers (Roche Diagnostics International Ltd., Rotkreuz, Switzerland). Fasting serum tyrosine (sTYR) and serum phenylalanine (sPHE) were analysed on acidified urine and serum samples from each visit, as previously described in both NAC and SN2 samples, using tandem mass spectrometry on the same analytical platform [14,15]. uUREA and uCREAT were photometrically assayed on a Roche Cobas 701 using an automated assay (hydrolysis with urease and subsequent oxidation of NADH). uUREA was used to objectively estimate dietary protein intake in keeping with other studies [16,17]. uCREAT was measured using a validated Jaffe reaction.

### 2.4. Nitisinone Therapy

Nitisinone was commenced in eligible patients after tests, including serum and 24 h urine collections, were completed both in the NAC and SN2 cohorts at the first visit. The daily dose of nitisinone was mainly 2 mg daily, with a few being on 5 mg and 10 mg doses since 2022 in the NAC and 10 mg in the SN2 cohort.

### 2.5. Dietetic Assessments and Management in the NAC

Patients underwent a global nutritional assessment, which included objective anthropometry (MUAC, muscle mass, %body fat, hand grip strength) and habitual nutritional intake using a 7-day food diary (energy and protein intake). Body composition assessment was undertaken using standard techniques [7,18]. Objective data included Mid Upper Arm Circumference (MUAC in cm) and nondominant arm grip strength (kg). A digital dynamometer (model MG4800, Marsden Weighing Scales, Ltd., Rotherham, UK) was used for all measurements, undertaken in a consistent sitting position, specifically chosen for its lightness for this patient group as part of a longitudinal approach. Weight, muscle mass, and %body fat were assessed using bioimpedance measurements employing the dual frequency Tanita scale model DC-430S MA instrument (Tanita Corporation, Tokyo, Japan). Height was taken using a wall-mounted stadiometer (Seca 222, Seca GmbH, Hamburg, Germany) that was accurate to the nearest 0.1 cm. Weight was taken in light clothing to the nearest 0.1 kg. Body mass index (BMI) was calculated as the ratio of weight in kg to the square of height in meters as defined by WHO [7,19]. All data, including the MUAC and grip strength readings, were compared to the previous publication from the NAC and changed over time during this longitudinal study. The data were collected and analysed by two experienced dieticians.

Habitual food intake was assessed using a 7-day diet diary, which was completed the week before study site visits based on the same methodology reported in the earlier publication. Data were coded using standardised techniques and entered in computerised analysis databases (Microdiet version 3, from 2012 to 2014) and nutritics (using Microdiet version 8 from 2015 onwards17). Total protein intake was presented as g/kg body weight [7].

The main goal of nutritional management in the NAC was to maintain sTYR as low as possible to minimise corneal keratopathy using a controlled protein intake. Additional goals were to mitigate the potential adverse effects of a reduced protein intake by maintaining BMI within the desirable range and maintaining muscle mass. There were planned dietary actions in terms of change in dietary protein intake based on sTYR measurements (Table S1).

### 2.6. Statistical Analysis

Continuous variables for metabolites uHGA_24_, sHGA, sTYR, and sNIT were compared using ANOVA with the Tukey–Kramer test for multiple comparisons using Instat™ GraphPad 3. Two-sided 95% confidence intervals corresponding to a two-sided 5% level of significance were used throughout the analyses. SONIA 2 analyses described here were post hoc. Linear regression was employed to assess relationships between groups of data (Instat™ GraphPad 3, Boston, MA, USA).

## 3. Results

The three groups of data (NAC, SN2 N−, and SN2 N+) were characterised by age, weight, BMI, sTYR, sPHE, uUREA, and uCREAT, which included 63 (males 41 and females 22) in the NAC and 138 (males 85 and females 53) in the SN2 groups. MUAC and body compositional analysis data (%body fat, muscle mass), grip strength, energy and protein intakes (the latter two from 7-day food diaries) were only collected in a subgroup of 42 NAC patients (males 28, females 14), which were followed up over four years.

### 3.1. Baseline Comparisons

At baseline, the comparison of age, body weight, BMI, and uCREAT was not significantly different in the NAC, SN2 N−, and SN2 N+ groups, even though uCREAT was lower by 9% in the NAC group compared with the SN2 N− and SN2 N+ groups. sTYR [mean (SD)] (µmol/L) was significantly lower by 4.6% in the NAC [62 (39) (*p* < 0.01) compared with the SN2 N− [65 (16)] and SN2 N+ [65 (15)] groups. Likewise, uUREA was significantly lower by 21.1% in the NAC [247 (97)] (mmol/day) (*p* < 0.01) compared with the SN2 N− [313 (92)] and SN2 N+ [312 (94)] groups. However, sPHE [mean (SD)] (µmol/L) was significantly higher by 12.6 in the NAC [63.6 (11.5)] (*p* < 0.05) compared with the SN2 N− [56.5 (9.5)] and SN2 N+ [56.8 (9.5)] groups (Table S2).

At baseline, MUAC, hand grip strength, %body fat, muscle mass, daily energy, and protein intake only available for the NAC group were like the previously published NAC data (Table S3).

### 3.2. Change in Anthropometric and Metabolic Data in the NAC, SN−, and SN+ Groups over Time (Table 1)

**Figure 1:**
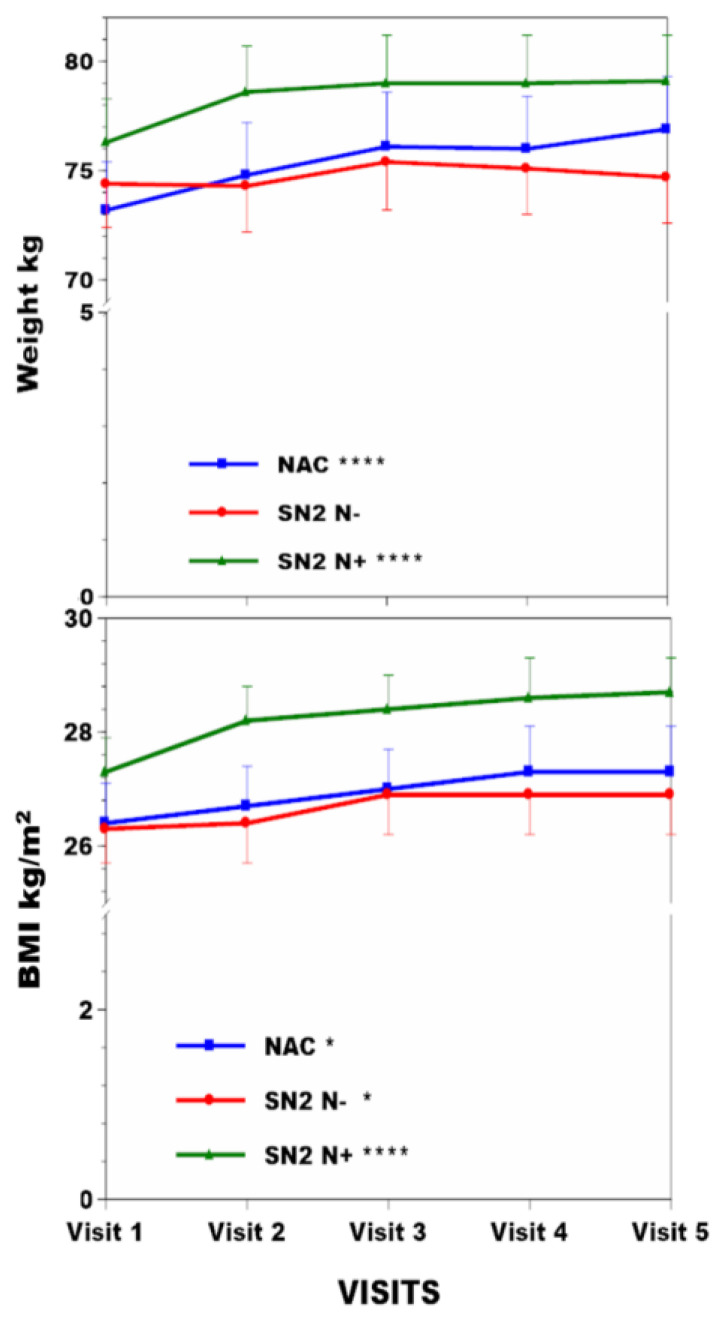
Weight and BMI changes across visits over four years are shown in NAC, SN2 N−, and SN2 N+. Note the use of *Y*-axis breaks. Statistical significance of differences across visits within each group (NAC or SN2 N− or SN2 N+) are indicated by *, **, ***, and ****, representing *p* < 0.05, 0.01, 0.001, and 0.0001.

**Figure 2:**
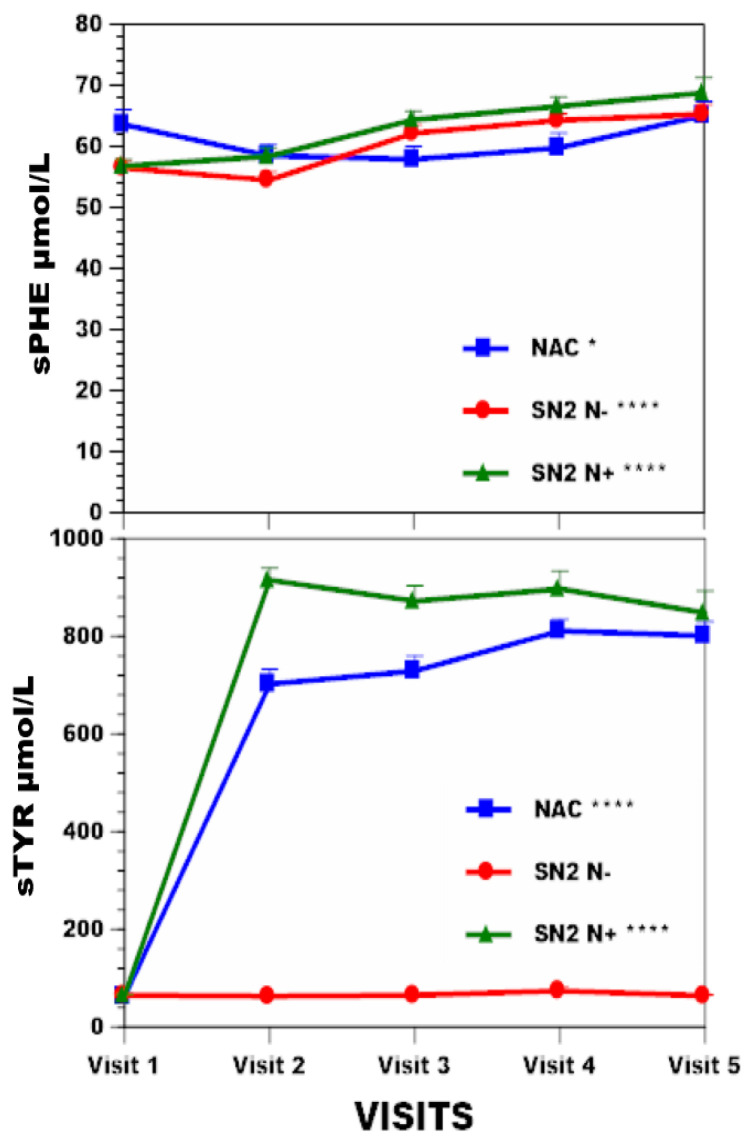
sTYR and sPHE changes across visits over four years are shown in NAC, SN2 N−, and SN2 N+. Note the use of *Y*-axis breaks. Statistical significance of differences across visits within each group (NAC or SN2 N− or SN2 N+) are indicated by *, **, ***and ****, representing *p* < 0.05, 0.01, 0.001, and 0.0001, respectively.

**Figure 3:**
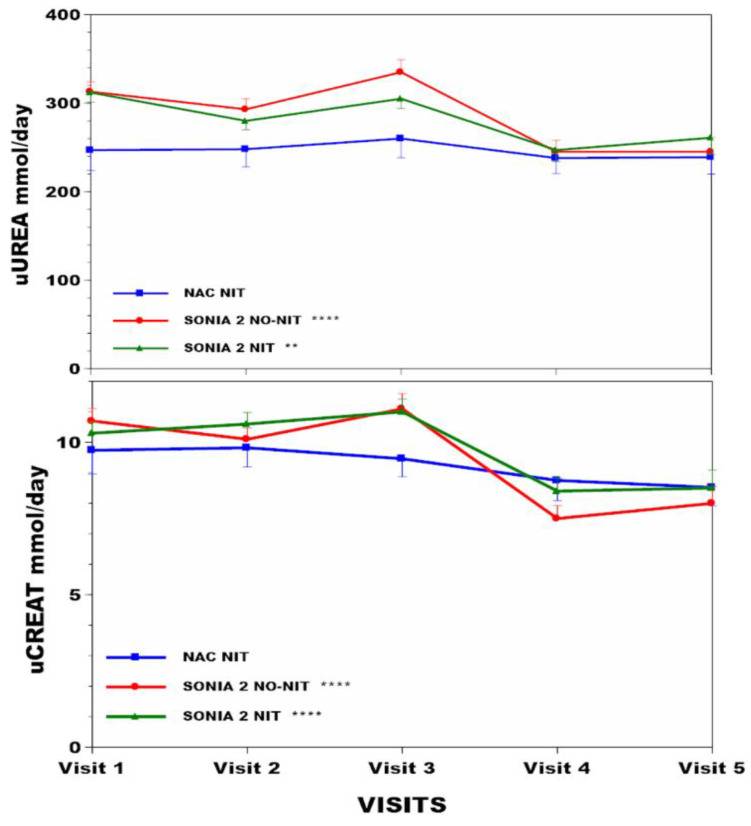
uUREA and uCREAT changes across visits over four years are shown in NAC, SN2 N−, and SN2 N+. Note the use of *Y*-axis breaks. Statistical significance of differences across visits within each group (NAC or SN2 N− or SN2 N+) are indicated by *, **, ***, and ****, representing *p* < 0.05, 0.01, 0.001, and 0.0001, respectively.

Weight increased by 5.1% (*p* < 0.001), 0.4%, and 3.7% (*p* < 0.001) in the NAC, SN2 N−, SN2 N+ groups, respectively (Table 1, Figure 1). BMI increased by 3.4% (*p* < 0.05), 2.3% (*p* < 0.05), and 5.1% (*p* < 0.001) in the NAC, SN2 N−, SN2 N+ groups, respectively (Figure 1). sTYR increased 11.9-fold (*p* < 0.001), −1.5%, and 12.1-fold (*p* < 0.001) in the NAC, SN2 N−, and SN2 N+ groups, respectively (Figure 2). There was no corneal keratopathy in the SN2 N− group, but 4.1% and 14.5%, respectively, in the NAC and SN2 N+ groups (Table 1). Change in sPHE in the NAC was minimal at 2.1% (*p* < 0.05), whereas sPHE increased by 15.6% (*p* < 0.001) and 21.1% (*p* < 0.001) in the SN2 N− and SN2 N+ groups, respectively (Figure 2). There was a smaller decrease in uCREAT of 12.5% in the NAC group (*p* < 0.05), with a more marked decrease of 25.2% (*p* < 0.001) and 17.5% (*p* < 0.001) in SN2 N− and SN2 N+, respectively (Figure 3). There was a minimal decrease in uUREA of 3.2% in the NAC, with a more marked decrease of 21.7% (*p* < 0.001) and 16.4% (*p* < 0.001) in SN2 N− and SN2 N+, respectively (Table 1, Figure 3).

**Table 1:** Anthropometric and metabolic data in the NAC, SN2 N−, and SN2 N+ groups across visits.

		Age (y)	Weight kg	BMI kg/m^2^	sTYR µmol/L	Corneal Keratopathy %	sPHE µmol/L	uCREAT mmol/day	uUREA mmol/day
NAC (*n* = 63)	Visit 1	47.1 (14.7)	73.2 (13.9) ***	26.4 (4.2) *	62 (39) ***		63.6 (11.5) *	9.74 (3.51) ^¥^	247 (97)
	Visit 2		74.8 (14.9)	26.7 (4.2)	702 (194)	4.1	58.5 (8.4)	9.82 (2.81)	248 (85)
	Visit 3		76.1 (15.5)	27 (4.2)	729 (200)		57.9 (9.6)	9.46 (2.65)	260 (91)
	Visit 4		76 (15.2)	27.3 (4.5)	811 (153)		59.8 (11.2)	8.75 (2.94)	238 (74)
	Visit 5		76.9 (15)	27.3 (4.5)	801 (185)		65.1 (11)	8.52 (2.68)	239 (81)
SN2 N− (*n* = 69)	Visit 1	47.6 (10.1)	74.4 (14.2)	26.3 (4.4) *	65 (16)		56.5 (9.5) ***	10.7 (3.4) ***	313 (92) ***
	Visit 2		74.3 (14.5)	26.4 (4.6)	63 (21)	0	54.5 (10.8)	10.1 (3)	293 (97)
	Visit 3		75.4 (15.3)	26.9 (4.9)	65 (14)		62.2 (9.9)	11.1 (3.8)	335 (110)
	Visit 4		75.1 (14.5)	26.9 (4.7)	74 (58)		64.3 (8.8)	7.5 (3.3)	245 (102)
	Visit 5		74.7 (14.9)	26.9 (4.8)	64 (16)		65.3 (11.9)	8 (4)	245 (102)
SN2 N+ (*n* = 69)	Visit 1	49.0 (11.3)	76.3 (15.1) ***	27.3 (4.4) ***	65 (15) ***		56.8 (9.5) ***	10.3(3) ***	312 (94) **
	Visit 2		78.6 (15.8)	28.2 (4.7)	915 (204)	14.5	58.4 (11.7)	10.6(3.1)	280 (83)
	Visit 3		79 (16)	28.4 (4.6)	872 (246)		64.4 (10.9)	11(3.4)	305 (92)
	Visit 4		79 (16.3)	28.6 (4.8)	897 (283)		66.6 (11.9)	8.4(3.3)	247 (100)
	Visit 5		79.1 (15.9)	28.7 (4.7)	848 (338)		68.8 (10)	8.5(4.4)	261 (137)

Values are shown as mean (SD). ^¥^ refers to *p* < 0.09; *, **, *** refer to *p* < 0.05, 0.01, and 0.001, respectively, for differences in the data within the group across visits.

### 3.3. Body Compositional Analysis in the NAC over Time

**Figure 4:**
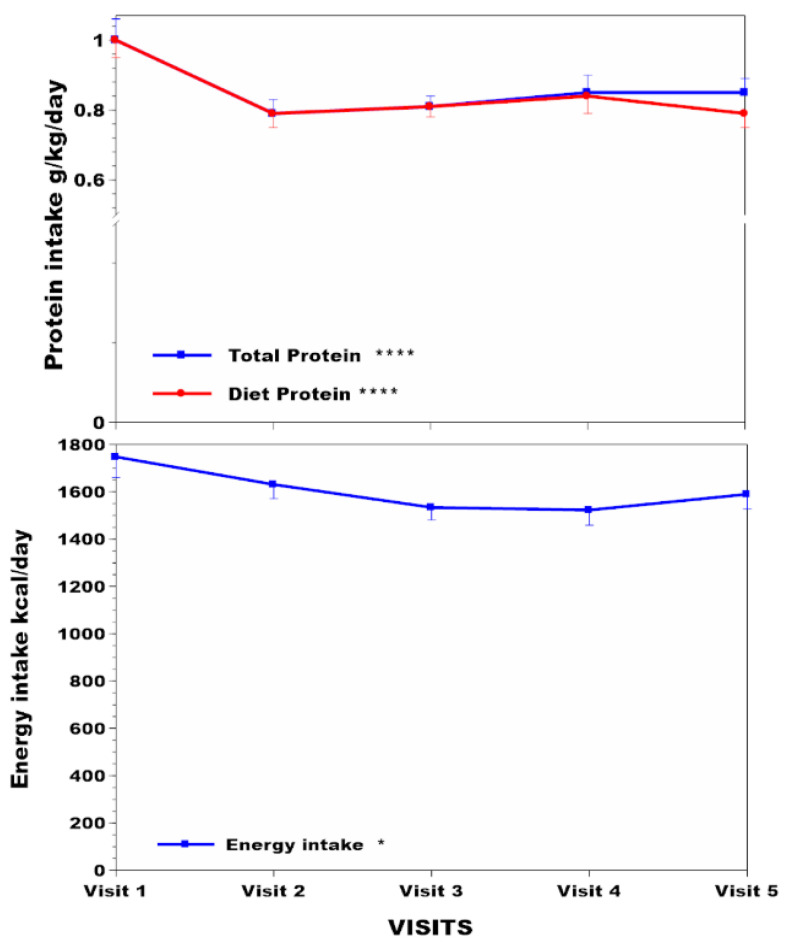
NAC protein and energy intake changes across visits over four years are shown in NAC. Note the use of *Y*-axis breaks. Statistical significance of differences across visits within each group is indicated by *, **, ***, and ****, representing *p* < 0.05, 0.01, 0.001, and 0.0001, respectively.

**Figure 5:**
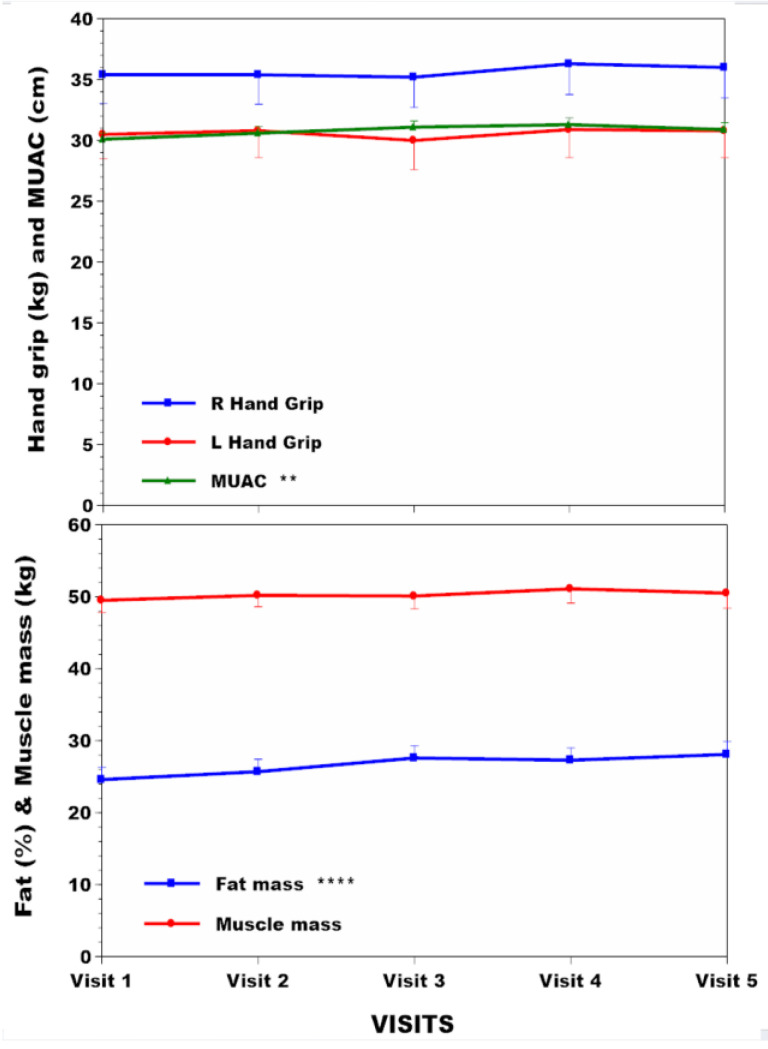
NAC hand grip, MUAC, %body fat, and muscle mass changes across visits over four years are shown in NAC. Statistical significance of differences across visits within each group is indicated by *, **, ***, and ****, representing *p* < 0.05, 0.01, 0.001and 0.0001, respectively.

The subgroup of 42 NAC patients had body compositional analysis data over time, which included 14 females and 28 males with respective mean (SD) ages of 47.9 (15.5) and 48.3 (13.3) years (Table S4). Dietary protein intakes without amino acid supplements decreased by 21% over the four years (*p* < 0.001) (Figure 4), while total protein intakes (calculated by adding dietary protein intake to amino acid supplements) showed a lesser decrease of 15% (*p* < 0.001) (Figure 4). The PHE/TYR-free amino acid supplementation varied from patient to patient and between visits depending upon sTYR values, making it difficult to give an exact figure for the number of patients receiving such supplements. Declared dietary energy intake in food diaries decreased over the study period by 9% (Figure 4) (*p* < 0.05). There were no decreases in the NAC subgroup over time with reference to hand grip strength and muscle mass (Figure 5). However, MUAC and %body fat increased significantly over the four years of study by 2.7% (*p* < 0.01) and 14.2% (*p* < 0.001), respectively (Table S4, Figure 5).

### 3.4. Linear Regression Analysis of Relationships between sTYR, Total Protein Intake, and uUREA with Other Measurements

Values greater than 400 µmol/L were chosen to study sTYR relationships with other groups of data so that compliance with nitisinone was satisfactory in these datasets. Examining the sTYR relationships, there was no relationship between uUREA and sTYR (R 0.08, *p* = 0.15) (Figure S2). There was a weakly positive but significant relationship between sPHE and sTYR (R 0.18, *p* < 0.001) (Table 2, Figure S3). There was no relationship between sTYR and dietary protein intake (R 0.04, *p* = 0.54) (Figure S4). Further, there was also no relationship between sTYR and total protein intake (R 0.1, *p* = 0.09) (Figure S5).

Examining the relationship between protein intake and sTYR in more detail, the relationship between estimated protein intake (based on uUREA) and sTYR was minimally positive in the NAC group (R 0.11; *p* = NS), whereas it achieved statistical significance in the SN2 N+ group (R 0.17; *p* < 0.01 (Figure S6a,b)). Estimated protein intake and sTYR in the combined NAC/SN2 N+ group changed over time and showed a marginally more positive relationship at 12 months (R 0.26; *p* < 0.01) than at 24 (R 0.21; *p* < 0.05), 36 (R 0.18; *p* = 07), and 48 (R −0.001; *p* = 99) months (Figure S6c–f)).

The regression relationship between total protein intake by food diary and estimated protein intake in the NAC was moderately positive (R 0.45; *p* < 0.0001) (Figure S7).

Total protein relationships were examined at baseline and months 12, 24, 36, and 48 data points in the NAC, SN2 N−, and SN2 N+ groups to maximise data comparisons. Examining the relationships of total protein intake with other data revealed a strong inverse relationship between body weight and total protein intake (R −0.57; *p* < 0.0001) (Figure S8), as there also was with BMI (−0.53, *p* < 0.0001) (Figure S9). Similar significant negative relationships were seen between total protein intake and muscle mass (R −0.39, *p* < 0.0001) (Figure S10), as well as with MUAC (R −0.48, *p* < 0.0001) (Figure S11). Interestingly, an increase in %body fat was seen with regards to a decrease in total protein intake (R −0.29, *p* < 0.0001) (Figure S12). Further, a weak inverse relationship between grip strength and protein intake was also noted (left-hand grip: R −0.14, *p* < 0.02; right-hand grip: R −0.12, *p* < 0.04) (Table 2, Figures S13 and S14).

The final relationship examined was between uUREA and uCREAT, which was strongly positive (R 0.69, *p* < 0.0001) (Table 2, Figure S15).

## 4. Discussion

Inhibition of HPPD by nitisinone markedly increases tyrosinaemia, requiring concomitant nutritional management [9]. The goals of nutritional management for nitisinone-induced tyrosinaemia are minimising tyrosinaemia, preventing the health consequences of tyrosinaemia, and maintaining optimal body weight, composition, and nutrition.

The NAC group employing nitisinone 2 mg showed the least increase in sTYR, unlike the SN2 N+ 10 mg nitisinone dose group. In the NAC and SN2 N+ groups, sTYR increased progressively, reflecting the sustained inhibition of HPPD and the continuing dietary protein restriction compliance challenge. Corneal keratopathy, consequent to tyrosine crystal formation in the cornea at high sTYR levels, was much greater in the SN2 N+, probably partly due to greater inhibition of HPPD at larger nitisinone dose, as well as a lack of individualised dietetic management. Pre-nitisinone sTYR accounted for less than 8% of tyrosine flux uncovered during nitisinone. Dietary PHE/TYR is needed for protein synthesis, as well as the synthesis of thyroid and catecholamine hormones and melanin. Flux in the PHE/TYR pathway is crucial to deal with surplus dietary PHE/TYR, with contributions from dietary PHE and TYR being around 60:40 [20,21].

In addition to nitisinone-related corneal keratopathy, other unwanted nitisinone effects included vitiligo in 5% of patients and unmasking, as well as worsening of Parkinson’s disease (PD) [22,23]. Cataracts, though prevalent before nitisinone, increased further during nitisinone therapy in the NAC despite dietetic management [24]. While the sTYR thresholds for corneal keratopathy are approximately defined at around 900 µmol/L, such thresholds have not been established for cataracts, vitiligo, or PD [25]. One publication showed a near 50% inhibition of tyrosine hydroxylase in the substantia nigra by increasing tyrosine concentrations to 200 µmol/L [26]. A considerably more restrictive dietary approach based on the long-term use of prescribed PHE/TYR-free protein substitutes would be necessary to manage these tyrosine-related non-corneal keratopathy health issues.

sPHE increased minimally in the NAC. A previous publication reported on the increase in sPHE during nitisinone therapy (i.e., SN2 N+ group) [13] but did not describe the no-nitisinone group (SN2 N− group). The previous publication attributed the increase in sPHE to the inhibition of phenylalanine hydroxylase by the nitisinone-induced tyrosinaemia [13] and the linear regression analysis of sPHE and sTYR in the combined NAC and SN2 N+ in the current analysis confirmed this relationship, supporting this explanation. A follow-up publication also found a marked increase in urine phenylalanine and associated metabolites in the SN2 N+ group, suggesting that surplus dietary PHE was being dealt with through urinary excretion as PHE metabolites during the inhibition of phenylalanine hydroxylase by tyrosinaemia while also minimising increase in sPHE [27].

Here, the no-nitisinone group (SN2 N−) was also examined and showed a similar increase in sPHE despite no nitisinone therapy. The mechanism discussed previously to account for the rise in sPHE during nitisinone cannot explain the increase in sPHE in the SN2 N− group, and indeed, the urinary PHE metabolite excretion paper also examined the SN2 N− group and found no increase in PHE urinary metabolites [27]. We suggest that protein restriction in the SN2 N− group resulted in a relative deficiency of PHE and other essential amino acids, leading to adaptations such as decreased oxidation, increased renal reabsorption and reduced general protein synthesis, thereby conserving PHE and increasing sPHE [28].

Dietary protein restriction is used to minimise tyrosinaemia. Remarkably, uUREA and sTYR show no statistically significant relationship. Dietary protein or total protein intakes adjusted per kg body weight and sTYR in the NAC showed no relationship. The reason for this lack of relationship between sTYR and protein intakes is puzzling. One explanation could be that there is still a vast excess of PHE/TYR within the whole-body amino acid pool, even with restricted protein intakes, evidenced by the appearance of new sTYR peri-nitisinone, as discussed already. The disconnect between food diary protein intake and sTYR may also be due to amino acid bioavailability due to lower protein digestibility from plant and animal sources since AKU patients are advised to consume less animal protein [29]. Other explanations may relate to a dose of nitisinone (with a larger dose correlating with a greater R coefficient in regression analysis) (Figure S6a,b), as well as adaptations over time (steeper regression slope at 12 months lost over month 48) (Figure S6c–f). Note that protein intakes decreased in the NAC from around 1g/kg body weight to a maximum of 0.8 g/kg body weight; dietary protein restriction beyond 75–80% may cause deficiency of tryptophan, methionine, threonine, and lysine. A PHE/TYR-free diet in mice made tyrosinaemic through nitisinone administration showed a remarkable resolution of tyrosinaemia [30], an impractical and risky approach in clinical management. We suggest instead that the state of the alternate metabolic pathways following HPPD inhibition, such as conversion to 4-hydroxyphenylpyruvate to 4-hydroxyphenyllactate, as well as PHE to TYR [20], may better explain the marked and variable increase in sTYR between patients [31] instead of variation in dietary protein intakes. Analysis of the relationship between sTYR and uUREA (surrogate for objective protein consumption) and subjective protein ingested per day are shown in Figures S16 and S17, describing potential pathophysiological considerations and management options.

At baseline, in the NAC group, body composition, MUAC, and other factors, such as hand grip strength, %body fat, muscle mass, daily energy, and protein intakes, resembled previously published data (Table S2), which attributed these lower values to malnutrition consequent to unsupervised attempts at lower protein intakes in AKU [7]. Another recent study reported on a nitisinone-treated AKU group in a long-term clinical trial and described a significant reduction in unsupervised protein intake, as well as biomarker change suggestive of lower lean body mass, although the no-nitisinone group (the designated SN2 N− in the current analysis) was not reported on [13].

A comparison of age, body weight, BMI, and uCREAT was not significantly different in the NAC, SN2 N−, and SN2 N+ groups at baseline, which was consistent with a similar nutritional status. Since the baseline nutritional state in the NAC group, described as malnutrition, was attributed to repeated attempts of self-directed dietary restriction, especially of protein, the similarity of baseline status in the non-UK SN2 populations could also signify malnutrition in these sedentary SN2 groups (Table S3) [7]. sTYR was significantly lower at baseline in the NAC (*p* < 0.01) compared with the SN2 N− and SN2 N+ groups, which could be due to greater dietary self-restriction in view of increased awareness of AKU as a disorder of protein metabolism due to the dissemination of such information by highly active patient charities in AKU especially in the UK, where the NAC is based [32]; most patients who participated in SN2 N– and SN2 N+ were from outside the UK and the NAC. This is supported by the finding of lower baseline uUREA, a biomarker for protein intake, in the NAC (*p* < 0.01) compared with the SN2 N− and SN2 N+ groups. Further, sPHE was significantly higher in the NAC at baseline (*p* < 0.05) compared with the SN2 N− and SN2 N+ groups even before nitisinone (Table S2); we suggest that this may reflect adaptations to the greater protein restriction in the NAC group as described earlier [20,28].

Supervised nutritional management only carried out in the NAC prevented further deterioration notably in muscle mass and strength despite protein restriction. The lack of improvement may be multifactorial including declining anabolic drive with ageing, increasing sedentary lifestyle consequent to debilitating musculoskeletal disease in AKU, continued protein restriction of around 20%, indicated by uUREA, requiring long-term adaptation.

Interestingly, even though there was a significant decrease in uCREAT in the NAC, there was no decrease in muscle mass or strength, which could be attributed to muscle protein metabolism adaptations. However, there is an alternative explanation. Creatinine is derived from creatine and has been used as a surrogate for muscle mass. Creatine can be synthesised endogenously in the body, and the conversion of this creatine to creatinine occurs within muscle at a constant daily rate. Creatine can also be obtained exogenously from the diet in ingested protein, especially cooked meats, and can contribute to uCREAT [33]. Since exogenous creatine intake decreases with protein restriction and changes in body creatine, and therefore, creatinine may not reflect only muscle mass. The strong linear regression analysis relationship described here between uUREA (estimated protein intake) and uCREAT is consistent with such a hypothesis (Figure S15). The discrepancy between lower uCREAT and stable muscle mass determination by bioimpedance in the present analysis also questions the use of uCREAT as a surrogate biomarker of muscle mass in prospective clinical trials [34,35].

Body weight, BMI, and %body fat increased in the NAC during nutritional management (Figure 1 and Figure 5). MUAC, a surrogate for BMI that does not differentiate between lean or fat mass, also increased. Protein may be the most satiating macronutrient [36], and its restriction may permit more fat and carbohydrate nutrients to be consumed instead, accounting for these %body fat changes in the NAC during the four years of follow-up. The inverse relationship between total protein intake and other indices, such as body weight, BMI, MUAC, and %body fat, supports this possibility (Figures S8–S12). Surprisingly, the weight and %body fat increase occurred despite an apparent decrease in total calories consumed (Figure 4). It is possible that subjective reporting via food diaries may underestimate intake, as claimed by other authors [37], although nothing is known about the energy expenditure in this situation. The moderate relationship between estimated protein intake (uUREA) and food diaries (R 0.45) in the current analysis supports underestimation in food dairies (Figure S7).

Weight gain was greatest at 5.06% in the NAC and lowest in SN2 N− (0.4%), where patients had the least incentive to reduce dietary protein, and intermediate in SN2 N+ (3.67%), where patients consistently restricted dietary protein on their own. Based on uUREA, protein restriction was successfully achieved in SN2 N− and SN2 N+, approximating 21.7 and 16.4%, respectively, whereas it was almost unchanged in the NAC (Table 1). uCREAT decreased least in the NAC, while SN2 N− and SN2 N+ showed greater decreases. This may indicate better muscle mass preservation in the NAC, where the algorithm-based dietary intervention included the use of amino acid supplements, unlike SN2, where this was not an option. Of note, direct confirmation of stable muscle mass was only possible in a subset of NAC patients.

There were limitations in this study. While temporal sequential body composition measurements offer insightful data not previously available, it should be noted that the body composition analysis in the NAC was available only in a subgroup; it should be noted, however, that this is the first study in AKU describing changes in body composition over time. Also, the body composition analyser was a dual-frequency model lacking the predictive accuracy of newer multi-frequency devices. While uUREA is a simpler measure, it cannot replace nitrogen balance studies, which require the measurement of nitrogen losses from urine, faeces, skin, sweat, and body secretions, but it is impractical in studies of this nature. Linear regression relationships described here are not necessarily causal. SN2 was a multiple-site study, unlike the NAC, even though every effort was made to harmonise study practices; on the other hand, data from multisite studies may be more beneficial and reliable. The dosages of nitisinone-inducing tyrosinaemia in NAC and SONIA 2 are different but unavoidable; a prior analysis showed the difference over four years between the doses in terms of tyrosinaemia is minor [38]. In a clinical trial like SN2, it was impractical to actively manage tyrosinaemia through specialist dietary management. AKU is a rare condition, making it a challenge to develop statistical power in any clinical study, even though this was one of the largest studies of its kind in this area.

## 5. Conclusions

In conclusion, the goals of nutritional management for nitisinone-induced tyrosinaemia in minimising tyrosinaemia and corneal keratopathy were probably achieved. Nutritional intervention succeeded in retaining muscle mass despite minimising protein to safe minimum recommendations without general prophylactic use of prescribed PHE/TYR-free products. Weight gain expressed as increased fat mass is a risk over the long term in this sedentary group. Tyrosinaemia during nitisinone is influenced more by the state of metabolic pathways in individual patients rather than being dominated by protein (PHE/TYR) intake. Dietetic intervention should continue until alternative therapies to manage tyrosinaemia are in place. The effects of protein-restricted diets described here go beyond their use in AKU and other inborn errors of metabolism. Further studies employing alternative methodologies, as well as longer-term studies, may be needed.

## Figures and Tables

**Table 2:** Linear regression relationships between sTYR, total protein intake, and uUREA with other parameters.

sTYR	Total Protein Intake	uUREA
Vs uUREA (R 0.08)	Vs BMI (R −0.53) ****	Vs uCREAT (R 0.69) ****
	Vs Weight (R −0.57) ****	
Vs sPHE (R 0.18) ***	Vs Muscle mass (R −0.39) ****	
Vs Diet protein intake (R 0.04)	Vs MUAC (R −0.48) ****	
Vs Total protein intake (R 0.1)	Vs %Body Fat (R −0.29) ****	
	Vs L Hand grip (R −0.14) *	
	Vs R Hand grip (R −0.12) *	

R reflects the linear regression coefficient, and *p* values are shown as *, **, ***, and ****, reflecting <0.05, <0.01, <0.001, and <0.0001, respectively. Each of the three columns describes relationships with other measurements.

## Data Availability

The original contributions presented in the study are included in the article/Supplementary Material, further inquiries can be directed to the corresponding author.

## Supplementary Materials

The following supporting information can be downloaded at https://www.mdpi.com/article/10.3390/nu16162722/s1.
