# Supplementary material for: Anthropometric, Body Composition, and Nutritional Indicators with and without Nutritional Intervention during Nitisinone Therapy in Alkaptonuria
Source: Nutrients. 2024 Aug 15;16(16):2722. doi: 10.3390/nu16162722 (PMC11357028; doi:10.3390/nu16162722)

Legend to Supplementary data

List of supplementary tables

Table S1. Nutritional management of tyrosinaemia during nitisinone in alkaptonuria in the NAC showing sTYR thresholds which trigger an appropriate dietary intervention action.

Table S2. Baseline body composition and dietary analysis in the NAC, SN2 N- and SN2 N+ groups (combined male and female groups). Values are shown as mean (SD). Statistical significance of differences across groups (NAC or SN2 N- or SN2 N+) are indicated by *, **, *** representing $p<0.05$, 0.01 and 0.001 respectively.

Table S3. Baseline body composition and dietary analysis in the NAC, SONIA 2 (SN2), and reference groups. Values are shown as mean (SD). Numbers of patients in NAC, SONIA 2 and reference population were 63, 138 and 74 respectively.

Table S4. Body composition and dietary analysis in the NAC cohort subgroup (n=42; males 28, females 14). Values are shown as mean (SD). Statistical significance of differences across visits over four years within each group (NAC or SN2 N- or SN2 N+) are indicated by *, **, *** representing $p<0.05$, 0.01 and 0.001 respectively.

List of supplemental figures

Figure S1. Metabolic pathway of phenylalanine/tyrosine highlighting the site of the enzyme defect observed in AKU and the site of action of nitisinone, a reversible competitive inhibitor of 4-hydroxyphenylpyruvate dioxygenase. The flux in the phenylalanine/tyrosine catabolic pathway shows that the ratio of flux from dietary PHE/TYR is around 60/40.

Figure S2. Linear regression relationship between sTYR(µmol/L) and uUREA (mmol/day) in combined NAC and SN2 N+ is shown. Only values of sTYR greater than 400 µmol/L were used in the analyses.

Figure S3. Linear regression relationship between sTYR (µmol/L) and sPHE (µmol/L) in combined NAC and SN2 N+ is shown. Only values of sTYR greater than 400 µmol/L were used in the analyses.

Figure S4. Linear regression relationship between sTYR (µmol/L) and Diet protein intake (g/kg/day) in combined NAC and SN2 N+ is shown.

Figure S5. Linear regression relationship between sTYR (µmol/L) and Total protein intake (g/kg/day) in combined NAC and SN2 N+ is shown.

Figure S6 a,b. Linear regression relationship between sTYR (µmol/L) and Estimated protein intake (g/kg/day) in the NAC (Figure S6a) and SN2 N+ (Figure S6b) are shown.

Figure S6 c-f. Linear regression relationship between sTYR (µmol/L) and Estimated protein intake (g/kg/day) in the combined NAC and SN2 N+ at month 12 (Figure S6c), month 24 (Figure S6d), month 36 (Figure S6e) and month 48 (Figure S6f) are shown.

Figure S7. Linear regression relationship between Estimated protein intake (g/kg/day) and total protein intake (Food diary) in the NAC is shown.

Figure S8. Linear regression relationship between Total protein intake (g/kg/day) and Body weight (kg) in combined NAC, SN2 N- and SN2 N+ is shown.

Figure S9. Linear regression relationship between Total protein intake (g/kg/day) and BMI (kg/M^2) in combined NAC, SN2 N- and SN2 N+ is shown.

Figure S10. Linear regression relationship between Total protein intake (g/kg/day) and Muscle mass (kg) in NAC is shown.

Figure S11. Linear regression relationship between Total protein intake (g/kg/day) and MUAC (cm) in NAC is shown.

Figure S12. Linear regression relationship between Total protein intake (g/kg/day) and % body fat in NAC is shown.

Figure S13. Linear regression relationship between Total protein intake (g/kg/day) and Left-hand grip strength (kg) in NAC is shown.

Figure S14. Linear regression relationship between Total protein intake (g/kg/day) and Right-hand grip strength (kg) in NAC is shown.

Figure S15. Linear regression relationship between uUREA (mmol/day) and uCREAT(mmol/day) in combined NAC, SN2 N- and SN2 N+ is shown.

Figure S16. Linear regression relationship between sTYR and uUREA describing their significance in terms of metabolic and management implications. A sTYR threshold of 800 µmol/L (midpoint of 700-900 µmol/L) and uUREA of 350 mmol/day (midpoint of laboratory reference range) (red dotted line at 500 mmol & green dotted line at 250 denote the upper & lower limits of laboratory reference range) were chosen for this illustration. Four zones were demarcated, and their significance indicated in the figure. Upper left zone represents high uUREA & acceptable sTYR indicating good metabolic compensation requiring only monitoring and support. Lower left zone represents low uUREA (restricted protein intake) and acceptable sTYR requiring only monitoring and support. Upper right zone represents higher uUREA & higher sTYR possibly requiring for dietary protein restriction and compliance training. Lower right zone indicates low uUREA (restricted protein intake) & high sTYR requiring PHE/TYR-free products

Figure S17. Linear regression relationship between sTYR and total protein intake describing their significance in terms of metabolic and management implications. A sTYR threshold of 800 µmol/L (midpoint of 700-900 µmol/L) and total protein intake of 1g/kg/day were chosen for this illustration. Four zones were demarcated, and their significance indicated in the figure. Upper left zone represents low protein intake with high sTYR requiring PHE/TYR-free products. Lower left zone represents low protein intake & acceptable sTYR requiring monitoring & support. Upper right zone represents higher protein intake & high sTYR requiring dietary protein restriction and compliance training. Lower right zone indicates high protein intake & acceptable sTYR indicating good metabolic adaptation and further monitoring and support.

Supplementary tables.

Table S1.

Table S1. Nutritional management of tyrosinaemia during nitisinone in alkaptonuria in the NAC		
Fasting sTYR (μmol/L)	Classification of tyrosinaemia	Action
<500	Minimal	No further action on 1g/kg/day protein intake
501-700	Mild	Reduce dietary protein to 0.9g/kg/day
701-900	Moderate	Reduce dietary protein to 0.8g/kg/day
>900	Severe	Consider phenylalanine/tyrosine-free amino acid supplements
>900 with Corneal keratopathy	Symptomatic	Stop nitisinone, and intensify dietary phenylalanine/tyrosine restrictions before restarting nitisinone

Table S2.

Table S2. Baseline body composition and dietary analysis in the NAC, SN2 N- and SN2 N+ groups (combined male and female groups)								
	Age (y)	Height cm	Weight kg	BMI kg/m^2	sTYR umol/L	sPHE umol/L	uCREAT mmol/day	uUREA mmol/day
NAC N+	47.1 (14.7)	165 (10.1)	73.2 (13.9)	26.4 (4.2)	62 (39)	63.6 (11.5)	9.74 (3.51)	247 (97)
SN2 N-	47.6 (10.1)	167.3 (9.5)	74.4 (14.2)	26.3 (4.4)	65 (16)	56.5 (9.5)	10.7 (3.4)	313 (92)
SN2 N+	49.0 (11.3)	166.3 (9.2)	76.3 (15.1)	27.3 (4.4)	65 (15)	56.8 (9.5)	10.3(3)	312 (94)
Values are shown as mean (SD)								

Table S3.

Table S3. Baseline body composition and dietary analysis in the NAC, SONIA 2 (SN2), and Reference groups						
	NAC		SONIA 2		Reference population ¥	
	Female	Male	Female	Male	Female	Male
Age yrs	50.1 (15.5)	46 (14.1)	49.2 (10.3)	47.8 (11)		
Height cm	157 (7.7)	170 (7.2)	159 (7.3)	171 (7.4)		
Weight kg	65.3 (20.9)	78.8 (15.4)	65.9 (14.7)	79.8 (12.9)		
BMI kg/m^2	26.3 (6.7)	27.2 (5.1)	25.8 (4.9)	27.2 (4.2)	26.3 (6.6)	26.9 (4.3)
MUAC cm	29.1 (4.6)	30.6 (3.4)			29.6 (4.7)	30.2 (3.2)
L Hand Grip kg	21 (6.8)	32.8 (11)			22.0 (9.2)	33.1 (13.3)
R Hand Grip kg	23.5 (7)	34.7 (11.8)				
%Body fat	30.8 (7.6)	24.2 (8.6)			29.5 (9.8)	24.8 (8.8)
Muscle mass kg	38.2 (3.8)	54.4 (6.6)			39.0 (4.4)	53.8 (6.5)
Energy intake kcal	1540 (404)	1818 (426)			1508 (368)	1857 (454)
Diet protein intake g/kg/day	0.96 (0.27)	0.99 (0.3)			1.0 (0.3)	1.0 (0.3)
sTYR umol/L	52.4 (13.9)	65.5 (40.9)	59.8 (14.8)	68.1 (14.4)		
sPHE umol/L	55 (6)	64.1 (11.6)	53.6 (8.8)	58.6 (9.4)		
uUREA mmol/day	186 (79.6)	290 (109)	273 (96.1)	337 (81)		
uCREAT mmol/day	8.04 (6)	10.4 (3.2)	8.4 (3)	11.8 (2.6)		

¥ refers to reference to adult AKU population (Judd et al, JMDR 2020;53:45-60)
Values are shown as mean (SD)

Table S4.

Table S4. Body composition and dietary analysis in the NAC cohort								
	MUAC cm	L Hand Grip Kg	R Hand Grip Kg	Muscle mass Kg	%Body Fat	Energy Kcal	Diet Protein g/kg/day	Total Protein g/kg/day
Visit 1	30.1 (3.2) **	30.5 (9.6)	35.4 (9.7)	49.5 (7.6)	24.6 (7.8) ***	1748 (505) *	1.0 (0.32) ***	1.0 (0.32) ***
Visit 2	30.6 (3.4)	30.8 (10.5)	35.4 (10.0)	50.2 (7.5)	25.7 (7.9)	1631 (347)	0.79 (0.22)	0.79 (0.22)
Visit 3	31.1 (3.1)	30.0 (11.3)	35.2 (10.2)	50.1 (8.1)	27.6 (7.8)	1534 (309)	0.81 (0.17)	0.81 (0.17)
Visit 4	31.3 (3.4)	30.9 (11.2)	36.3 (10.4)	51.1 (9.1)	27.3 (8.0)	1523 (375)	0.84 (0.29)	0.85 (0.29)
Visit 5	30.9 (3.5)	30.8 (10.6)	36.0 (10.3)	50.5 (9.5)	28.1 (1.8)	1590 (362)	0.79 (0.22)	0.85 (0.26)
*, **, *** refers tp p<0.05, 0.01 and 0.001 respectively for differences in the data within the group across visits<br>Values are shown as mean (SD)								

Supplementary figures.

Figure S1.

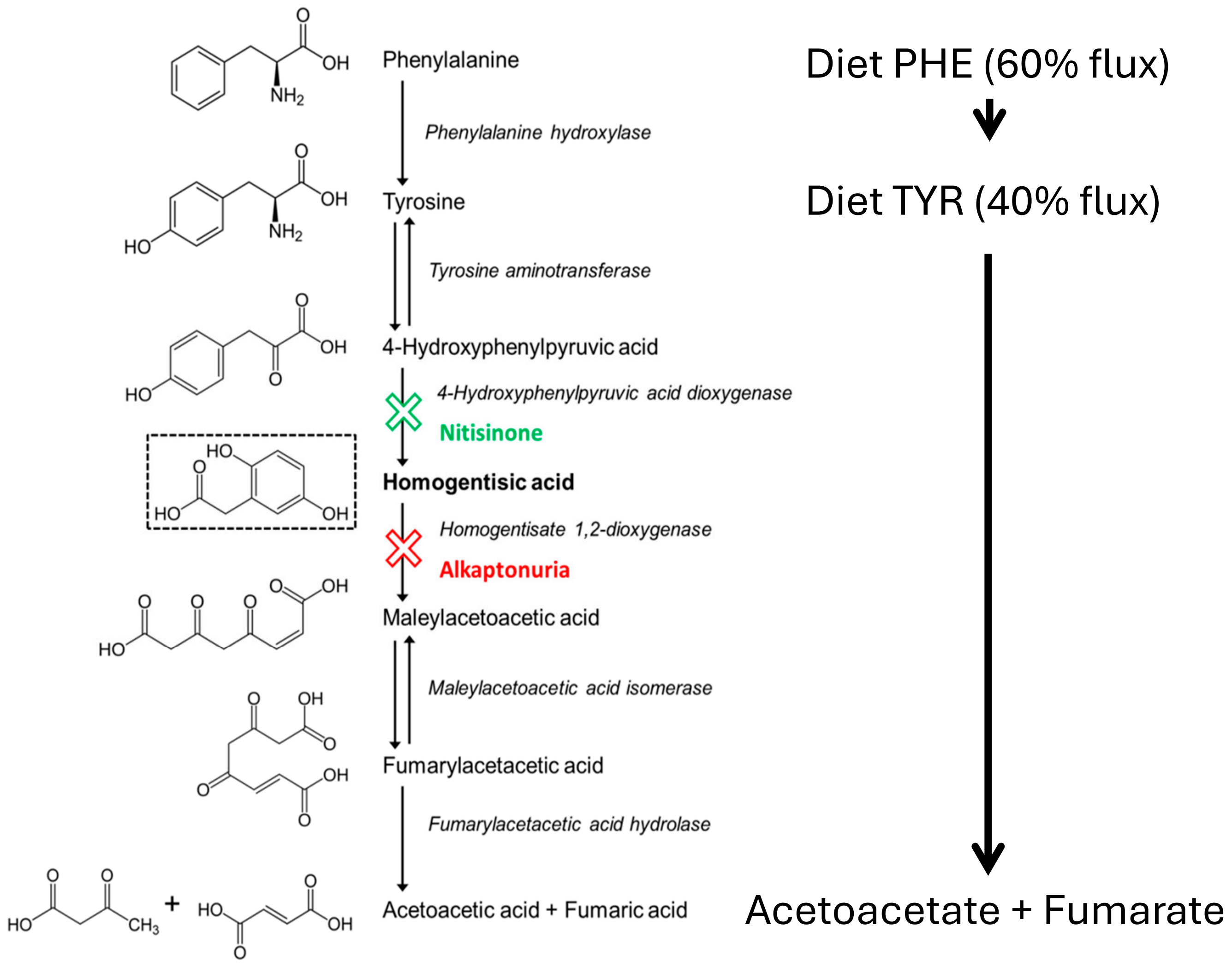

Figure S2.

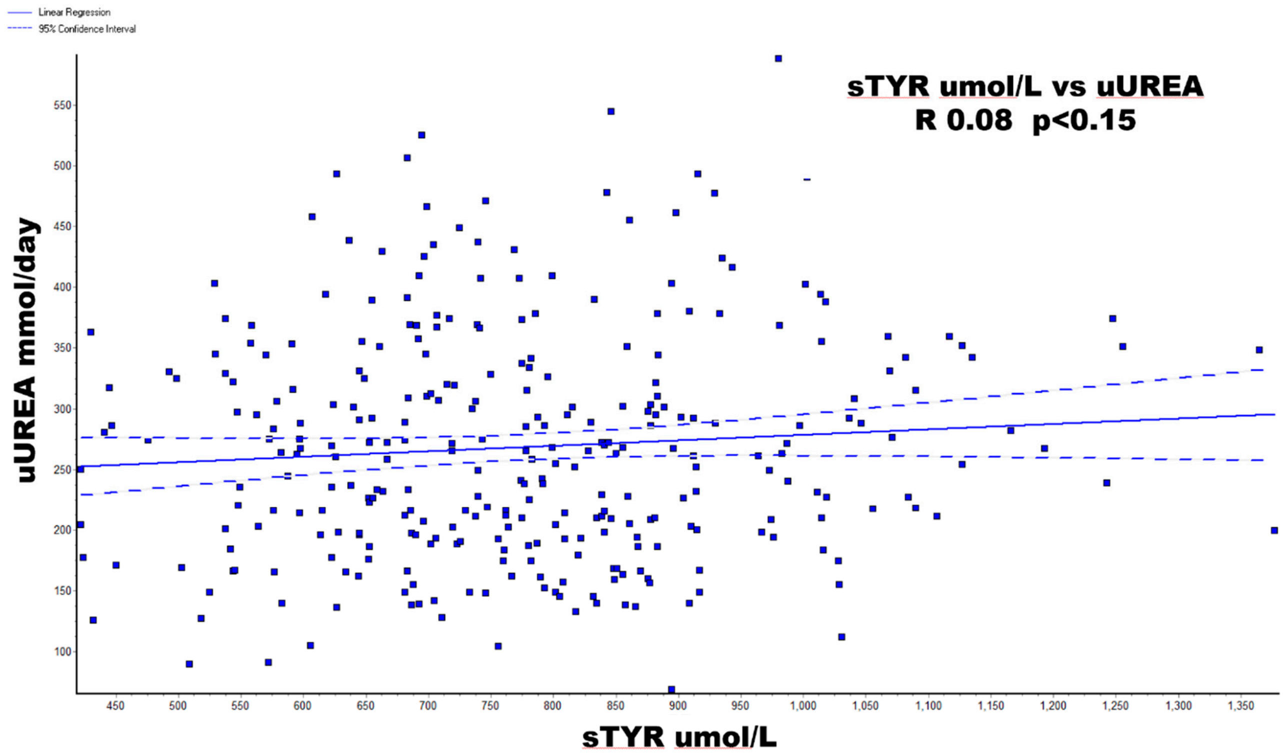

Linear Regression
95% Confidence Interval
sTYR umol/L vs uUREA
R 0.08 p<0.15
uUREA mmol/day
sTYR umol/L

Figure S3.

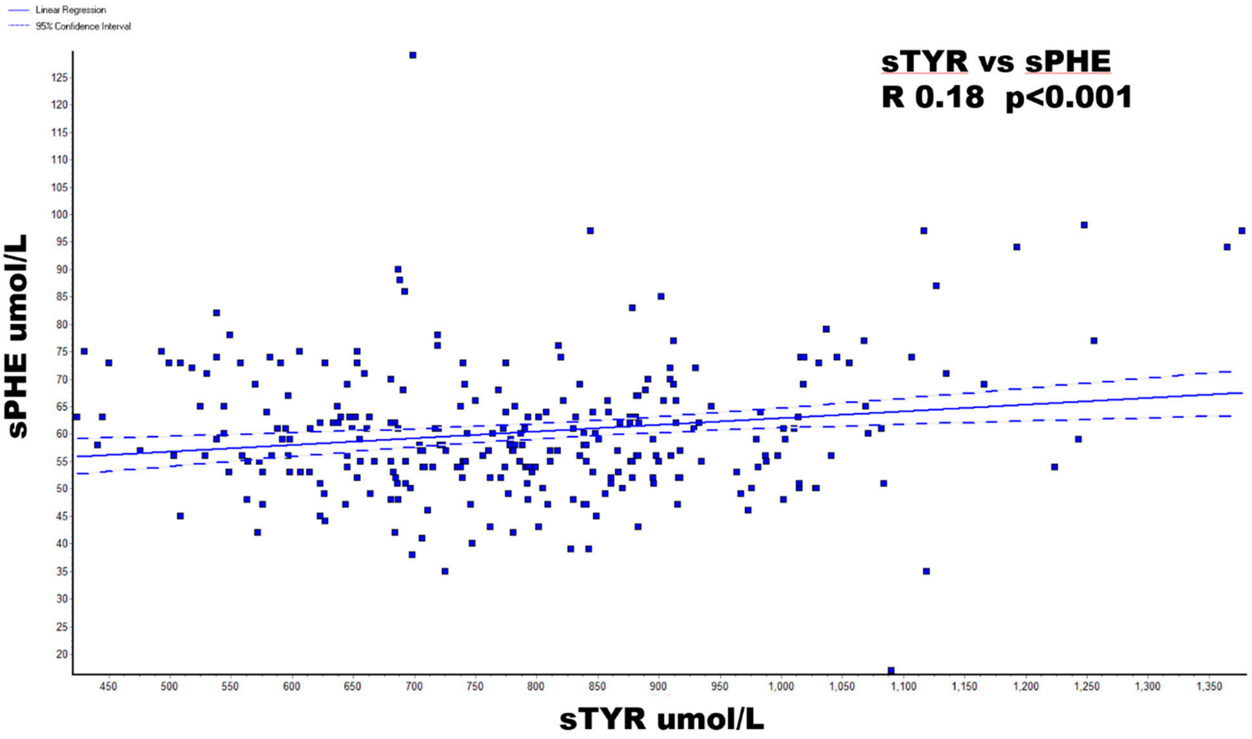

Linear Regression
95% Confidence Interval
sTYR vs sPHE
R 0.18 p<0.001
sPHE umol/L
sTYR umol/L

Figure S4.

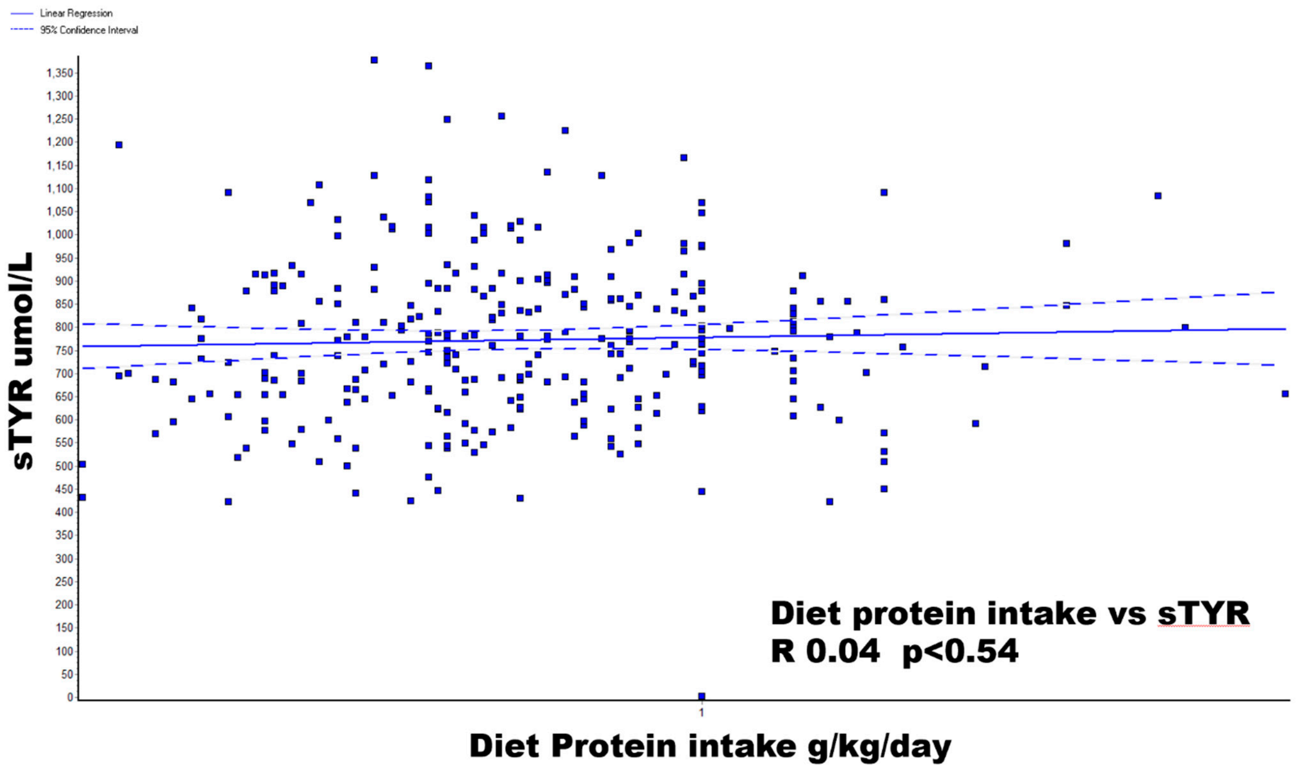

Figure S5.

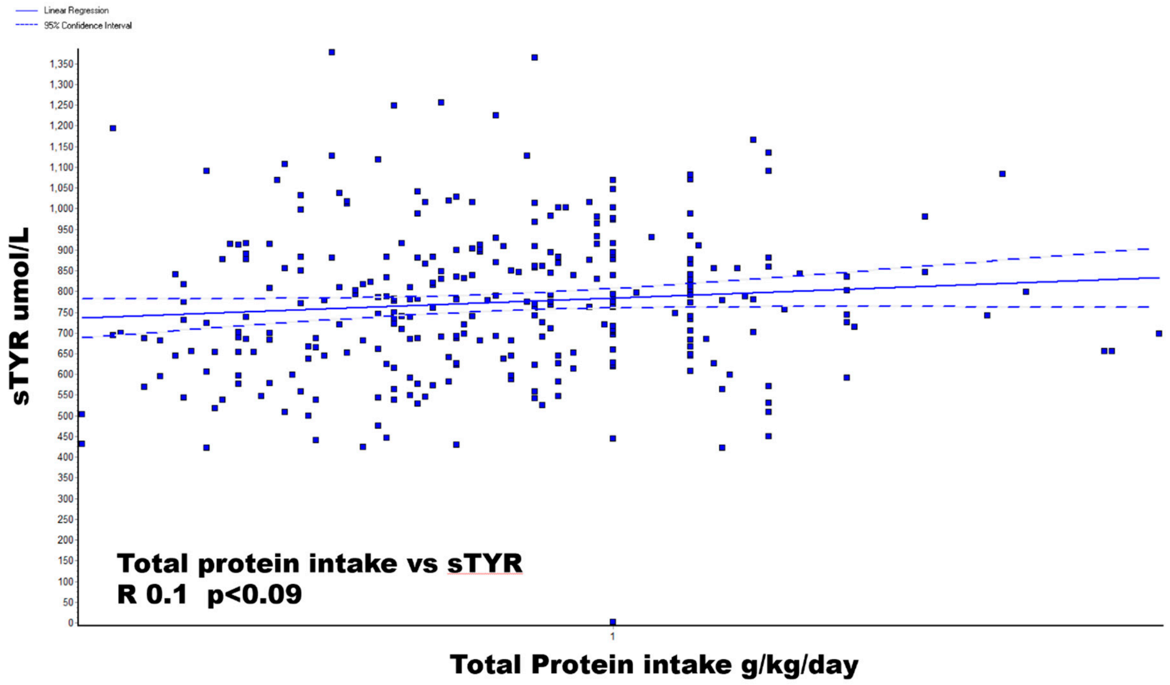

Figure S6 a,b.

(a)

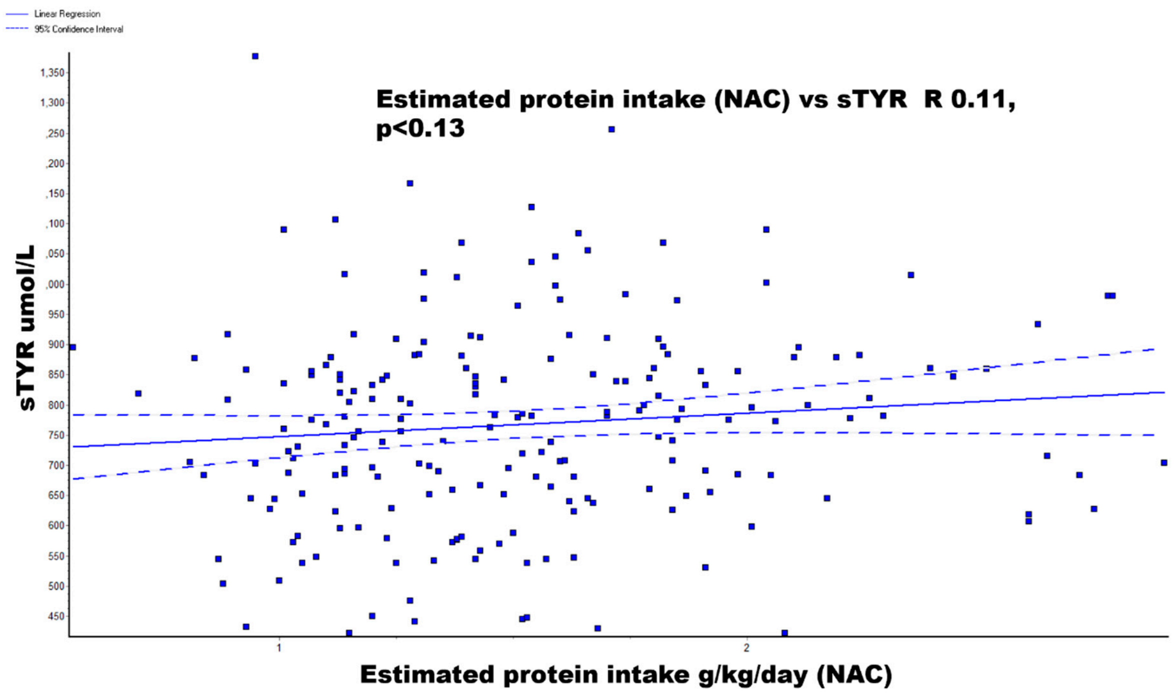

(b)

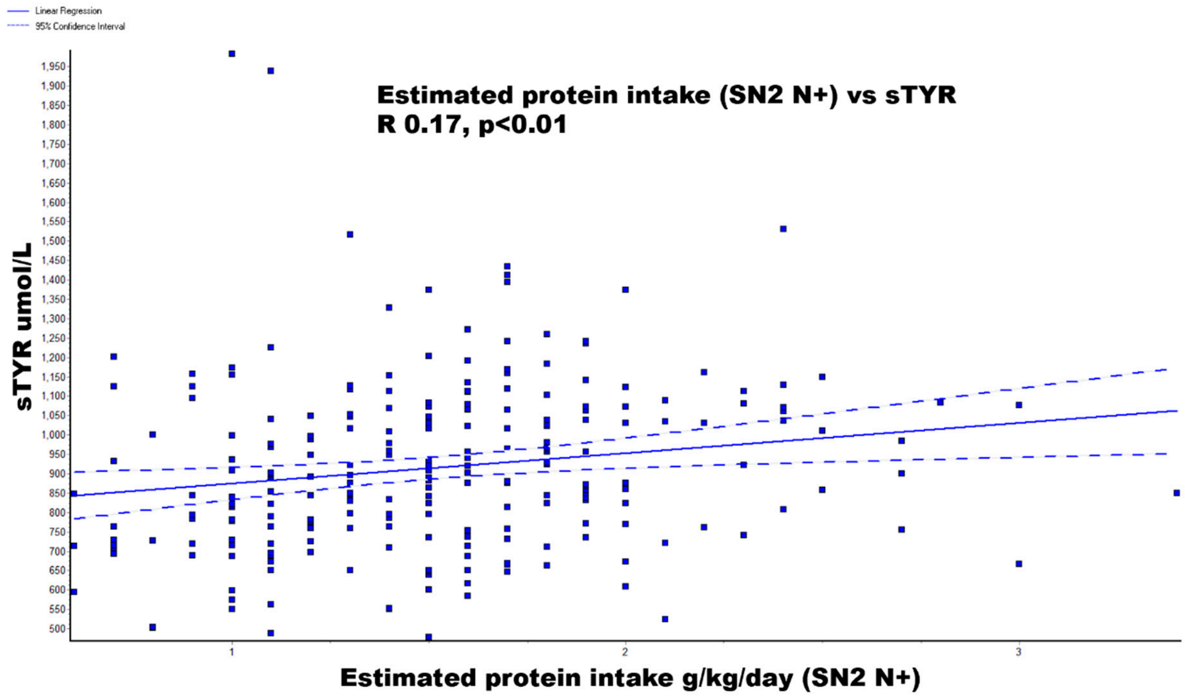

Figure S6c.

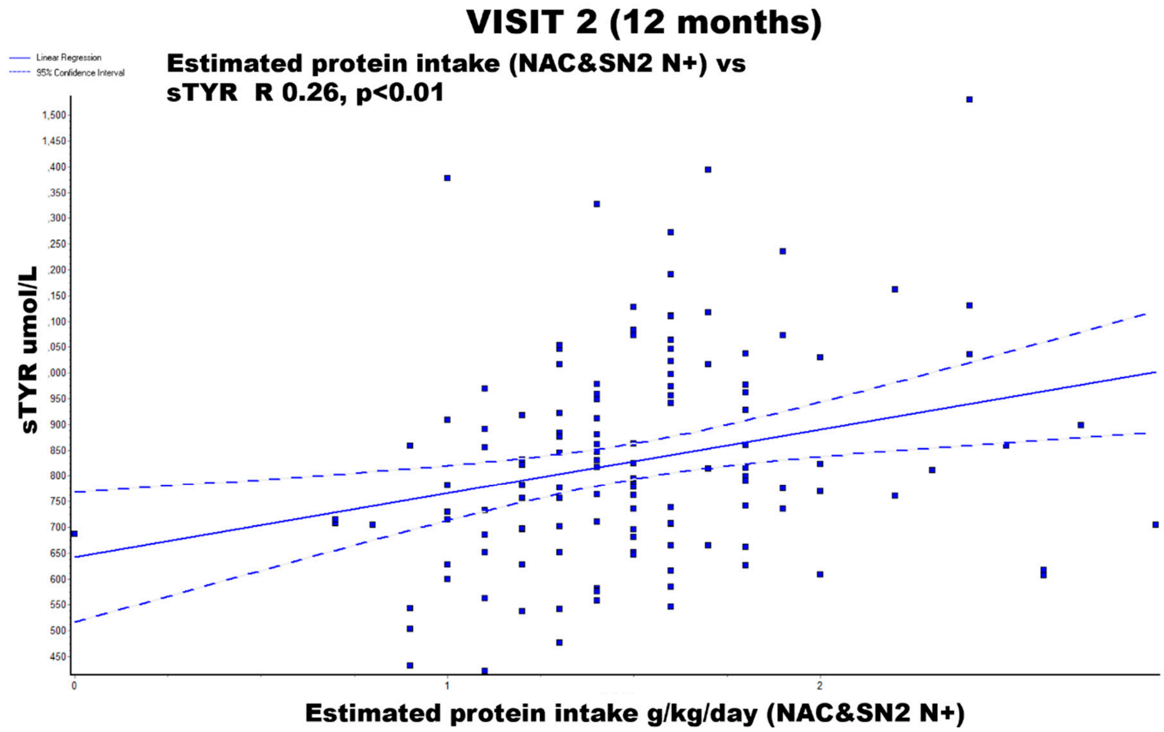

VISIT 2 (12 months)
Linear Regression
95% Confidence Interval
Estimated protein intake (NAC&SN2 N+) vs
sTYR R 0.26, p<0.01
sTYR umol/L
Estimated protein intake g/kg/day (NAC&SN2 N+)

Figure S6d.

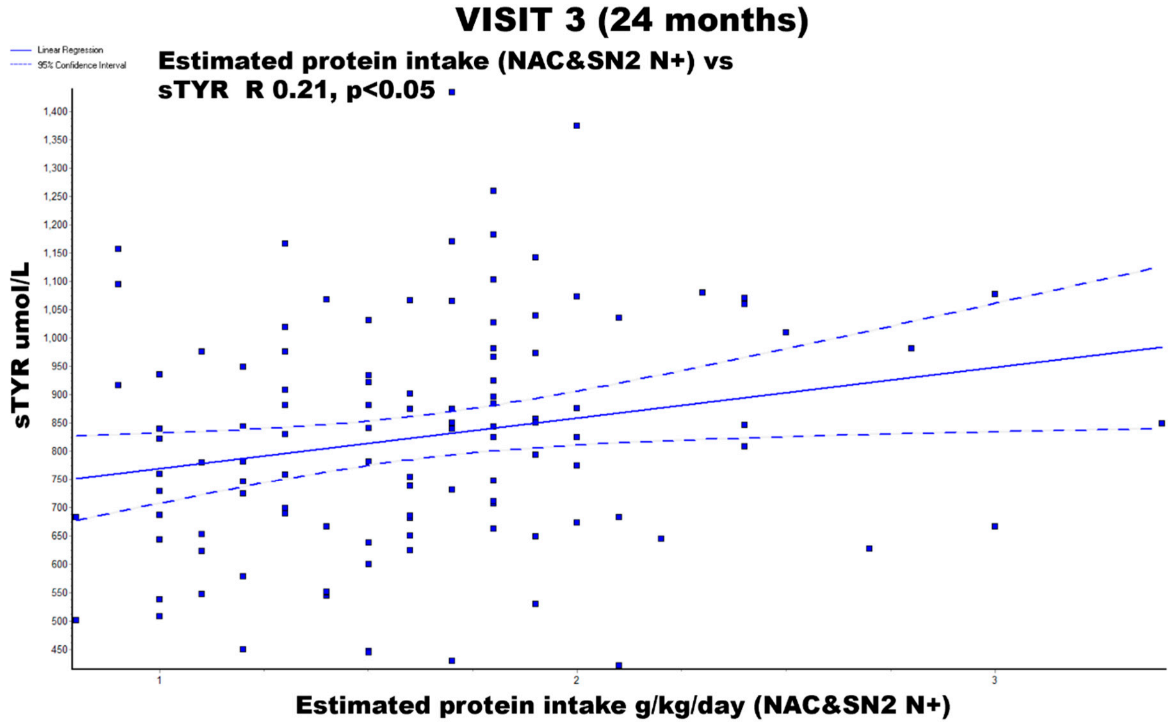
VISIT 3 (24 months)
Linear Regression
95% Confidence Interval
Estimated protein intake (NAC&SN2 N+) vs
sTYR R 0.21, p<0.05
sTYR umol/L
1,400
1,350
1,300
1,250
1,200
1,150
1,100
1,050
1,000
950
900
850
800
750
700
650
600
550
500
450
1
2
3
Estimated protein intake g/kg/day (NAC&SN2 N+)

Figure S6e.

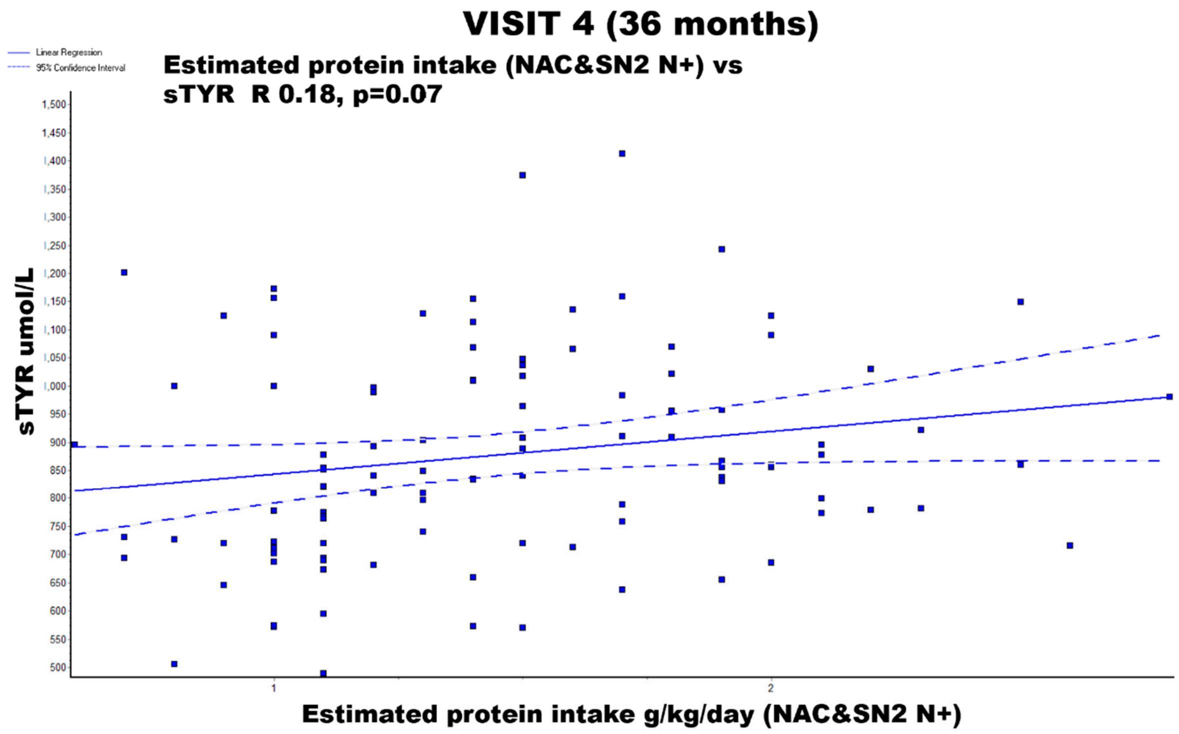
VISIT 4 (36 months)
Linear Regression
95% Confidence Interval
Estimated protein intake (NAC&SN2 N+) vs
sTYR R 0.18, p=0.07
1,500
1,450
1,400
1,350
1,300
1,250
1,200
1,150
1,100
1,050
1,000
950
900
850
800
750
700
650
600
550
500
sTYR umol/L
1
2
Estimated protein intake g/kg/day (NAC&SN2 N+)

Figure S6f.

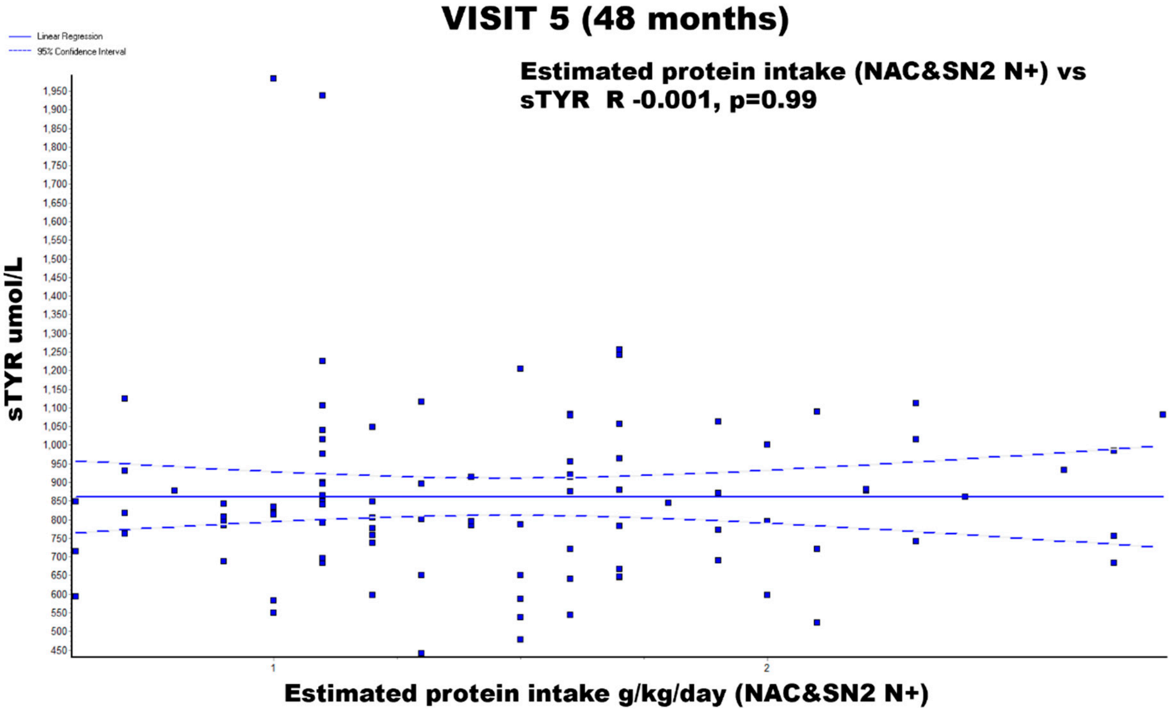
VISIT 5 (48 months)
Linear Regression
95% Confidence Interval
Estimated protein intake (NAC&SN2 N+) vs sTYR R -0.001, p=0.99
sTYR umol/L
1,950
1,900
1,850
1,800
1,750
1,700
1,650
1,600
1,550
1,500
1,450
1,400
1,350
1,300
1,250
1,200
1,150
1,100
1,050
1,000
950
900
850
800
750
700
650
600
550
500
450
1
2
Estimated protein intake g/kg/day (NAC&SN2 N+)

Figure S7.

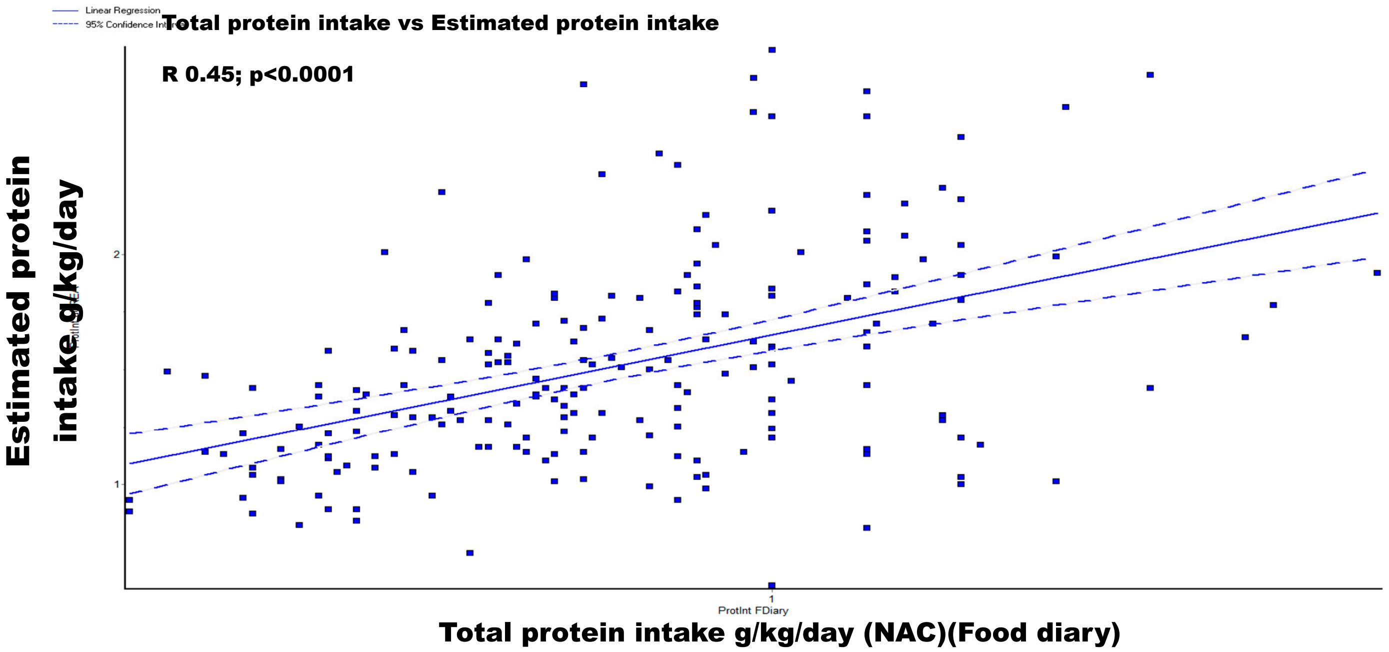
Linear Regression
95% Confidence Interval
Total protein intake vs Estimated protein intake
R 0.45; p<0.0001
Estimated protein intake g/kg/day
Total protein intake g/kg/day (NAC)(Food diary)
ProtInt FDiary
1
2
1

Figure S8.

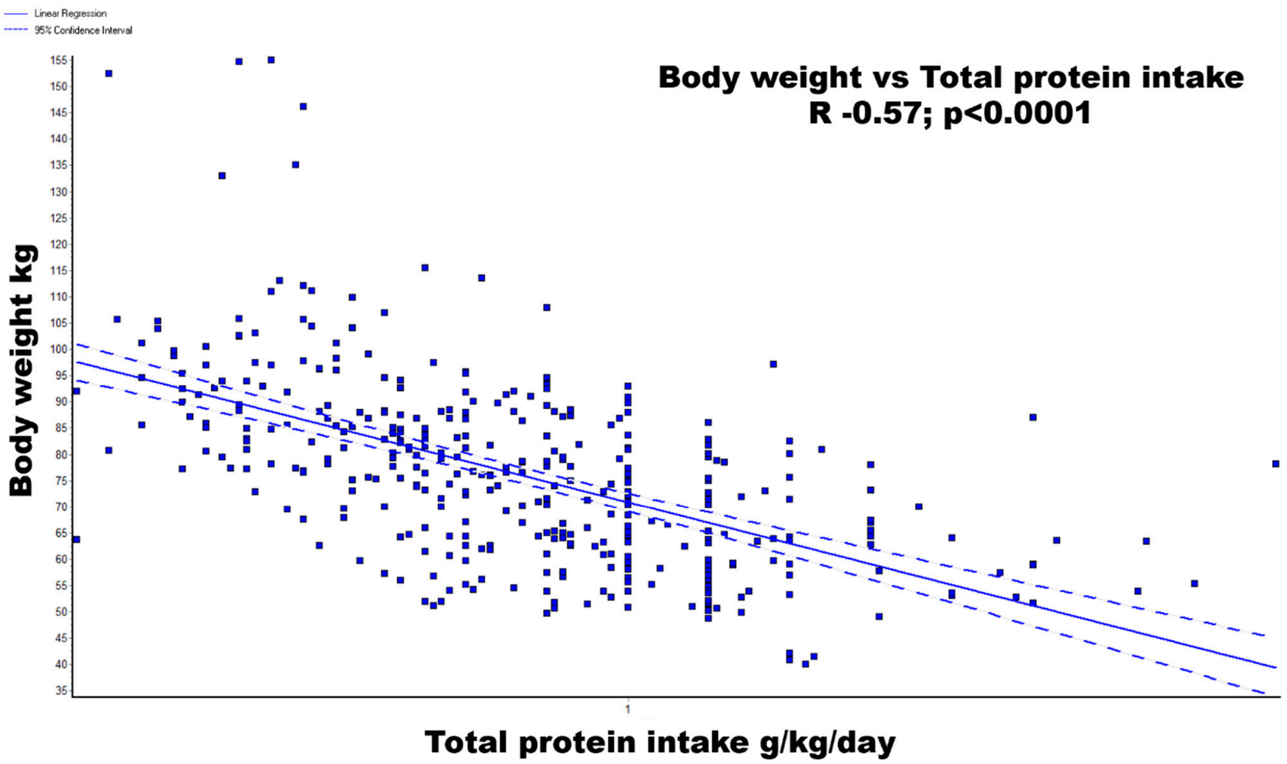

Figure S9.

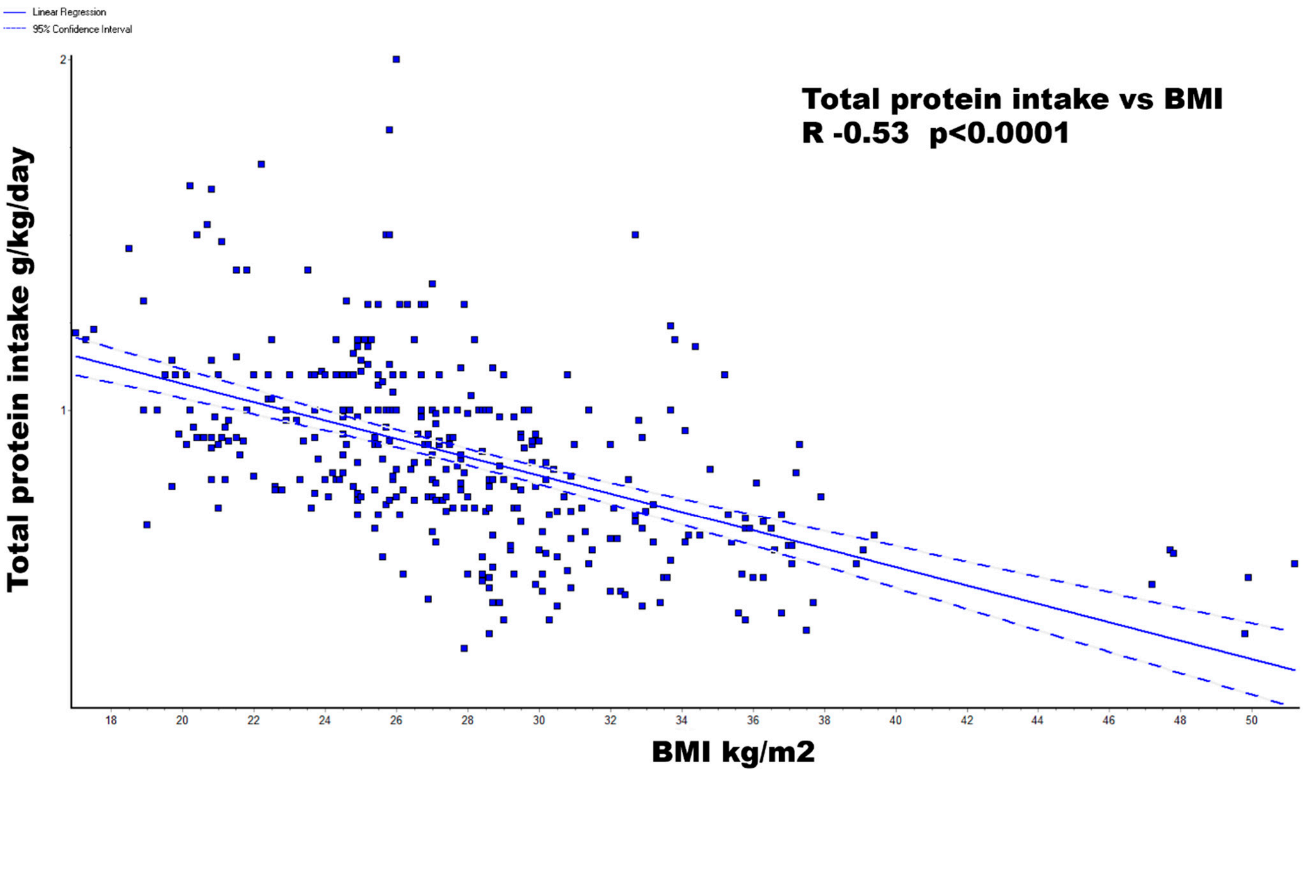

Figure S10.

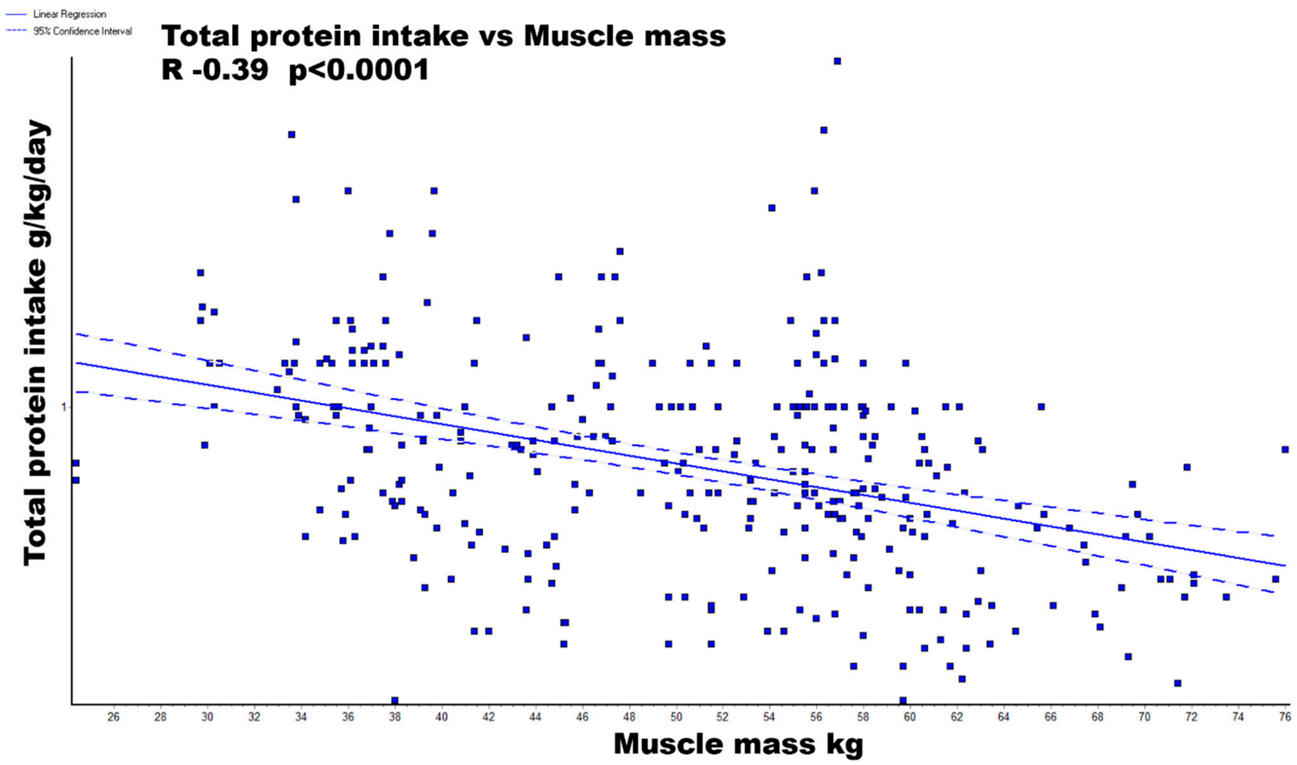

Figure S11.

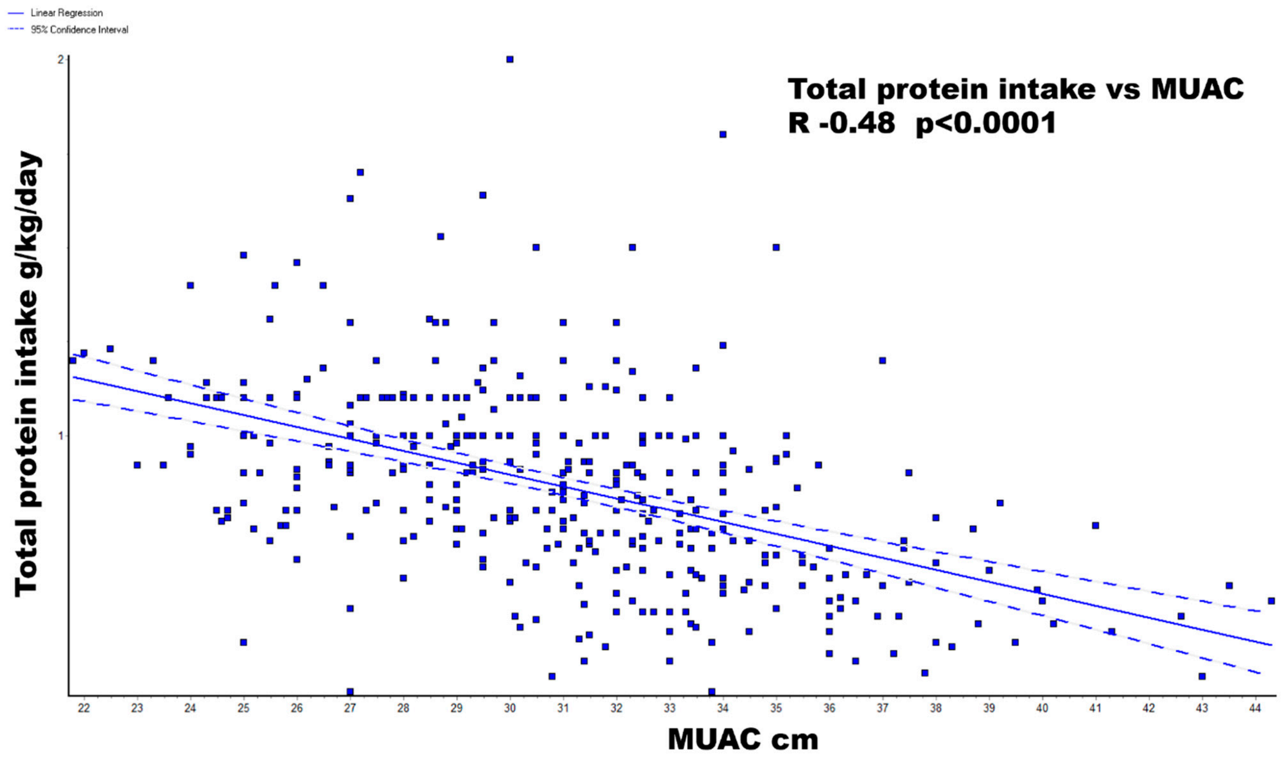

Figure S12.

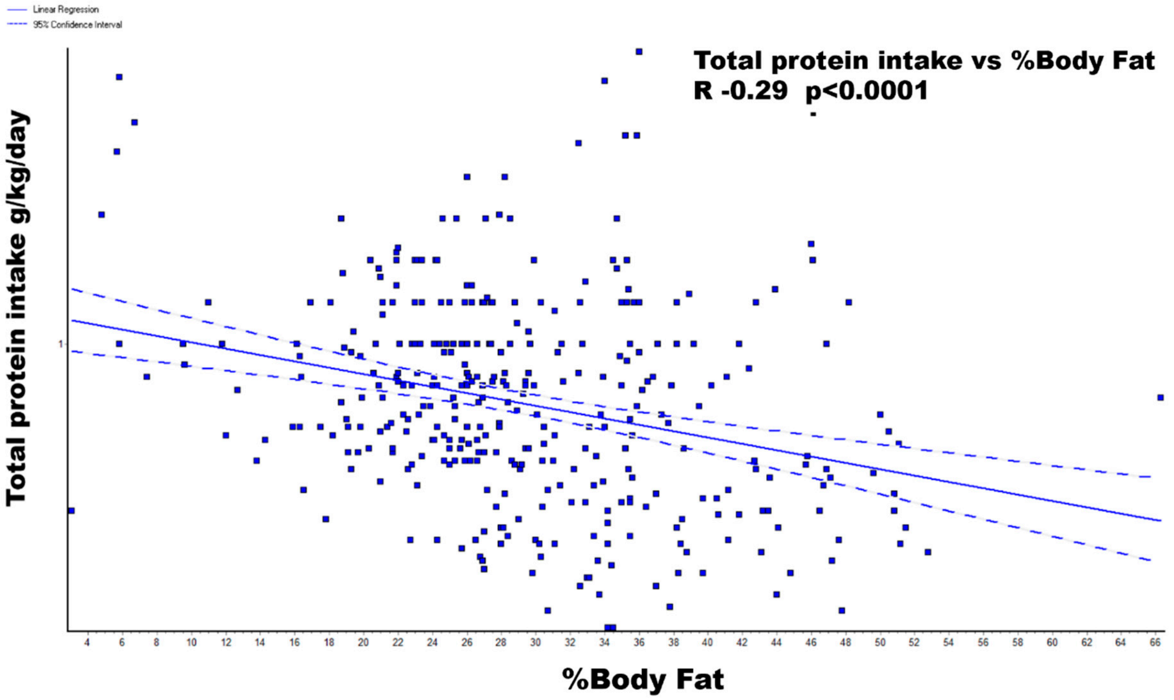

Linear Regression
95% Confidence Interval
Total protein intake vs %Body Fat
R -0.29 p<0.0001
Total protein intake g/kg/day
1
4
6
8
10
12
14
16
18
20
22
24
26
28
30
32
34
36
38
40
42
44
46
48
50
52
54
56
58
60
62
64
66
%Body Fat

Figure S13.

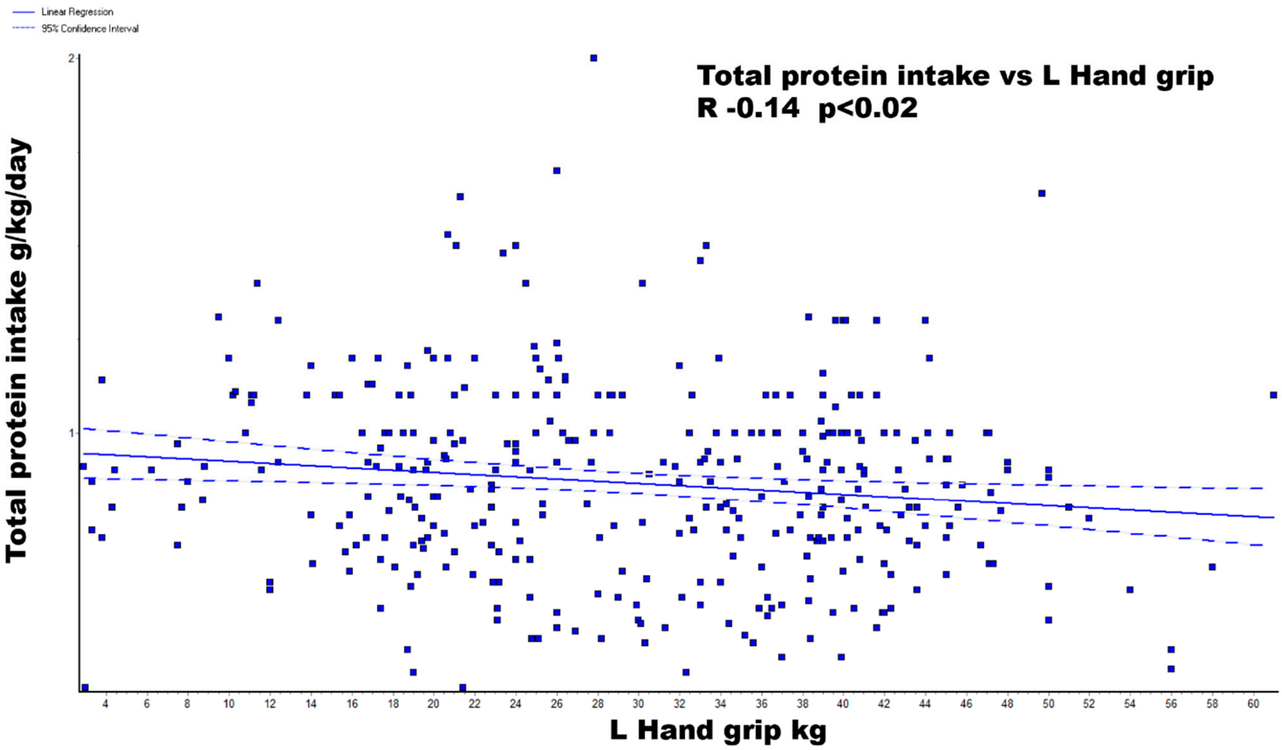

Figure S14.

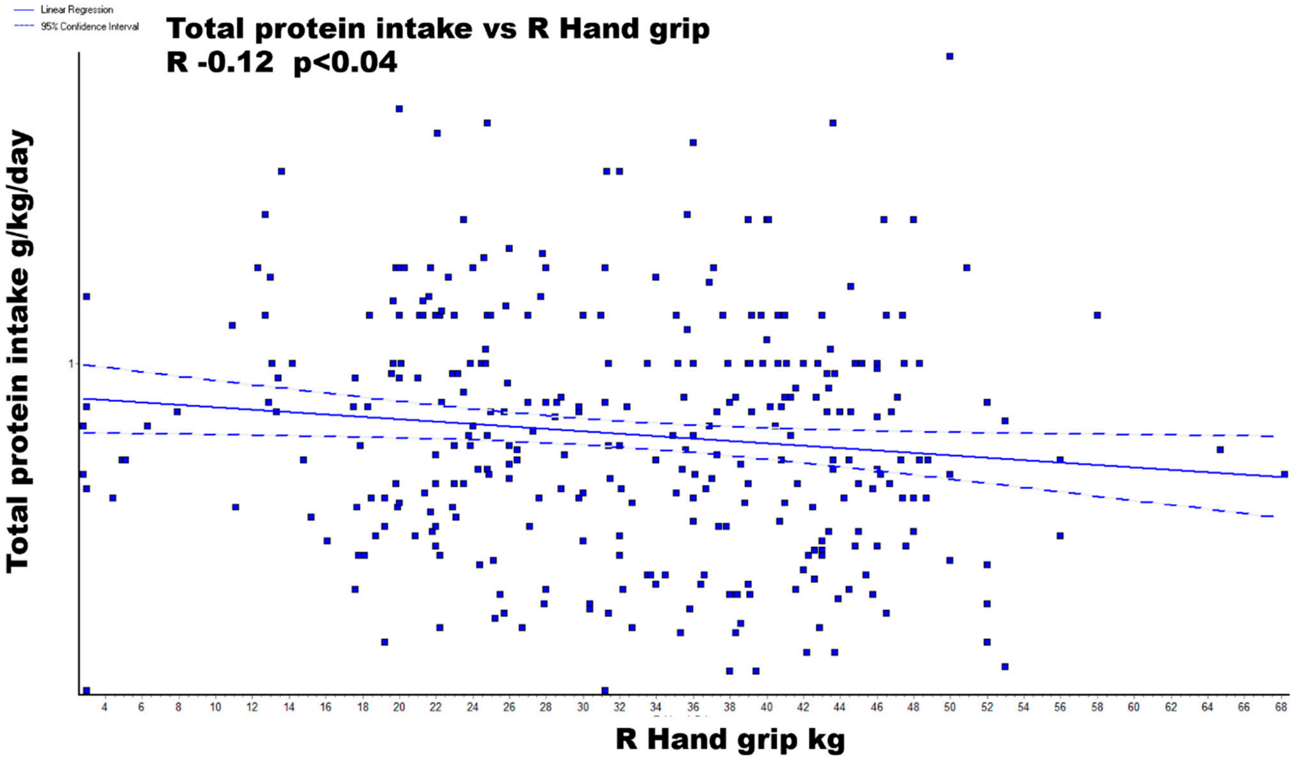

Figure S15.

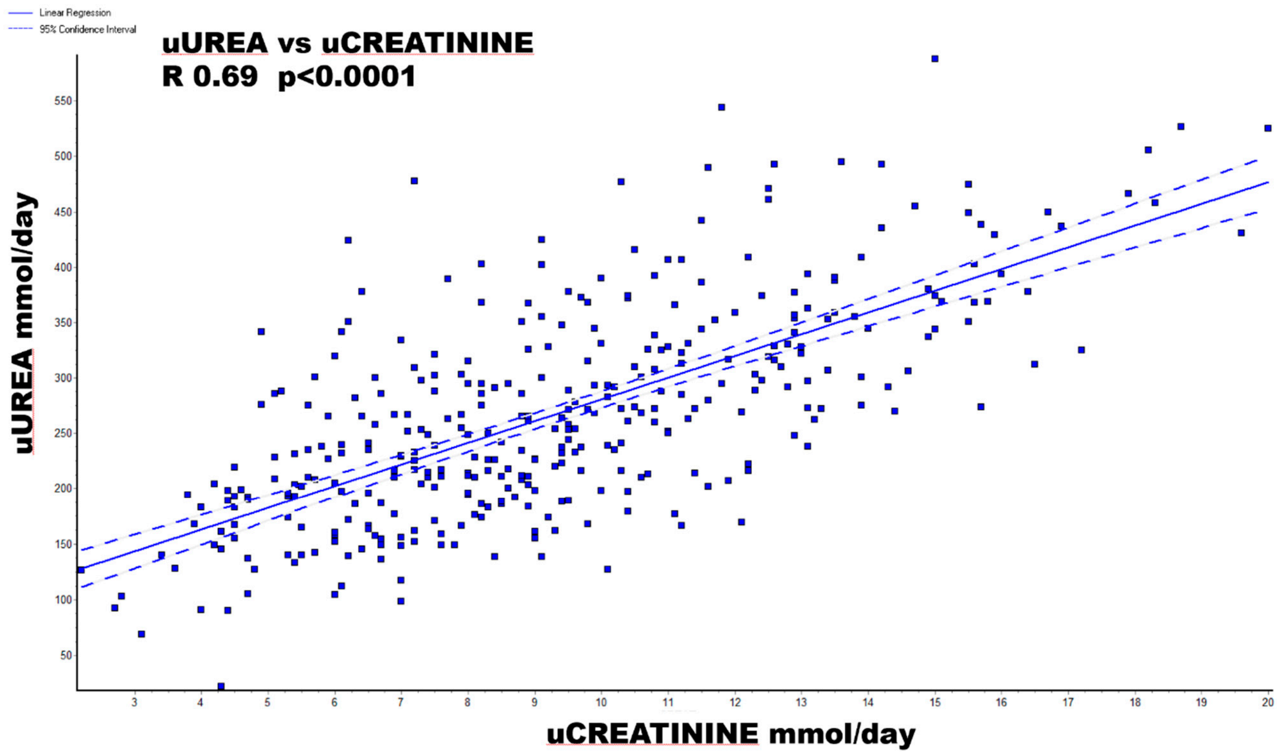

Linear Regression
95% Confidence Interval
uUREA vs uCREATININE
R 0.69 p<0.0001
uUREA mmol/day
uCREATININE mmol/day
550
500
450
400
350
300
250
200
150
100
50
3
4
5
6
7
8
9
10
11
12
13
14
15
16
17
18
19
20

Figure S16.

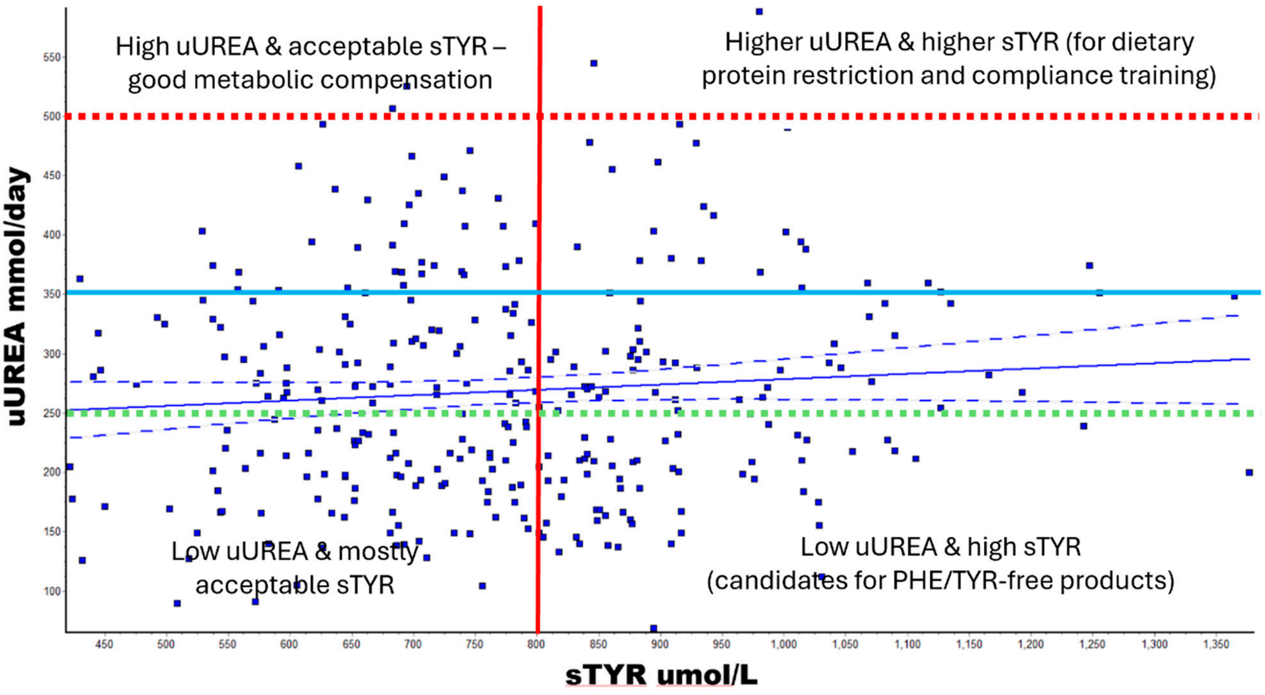

Figure S17.

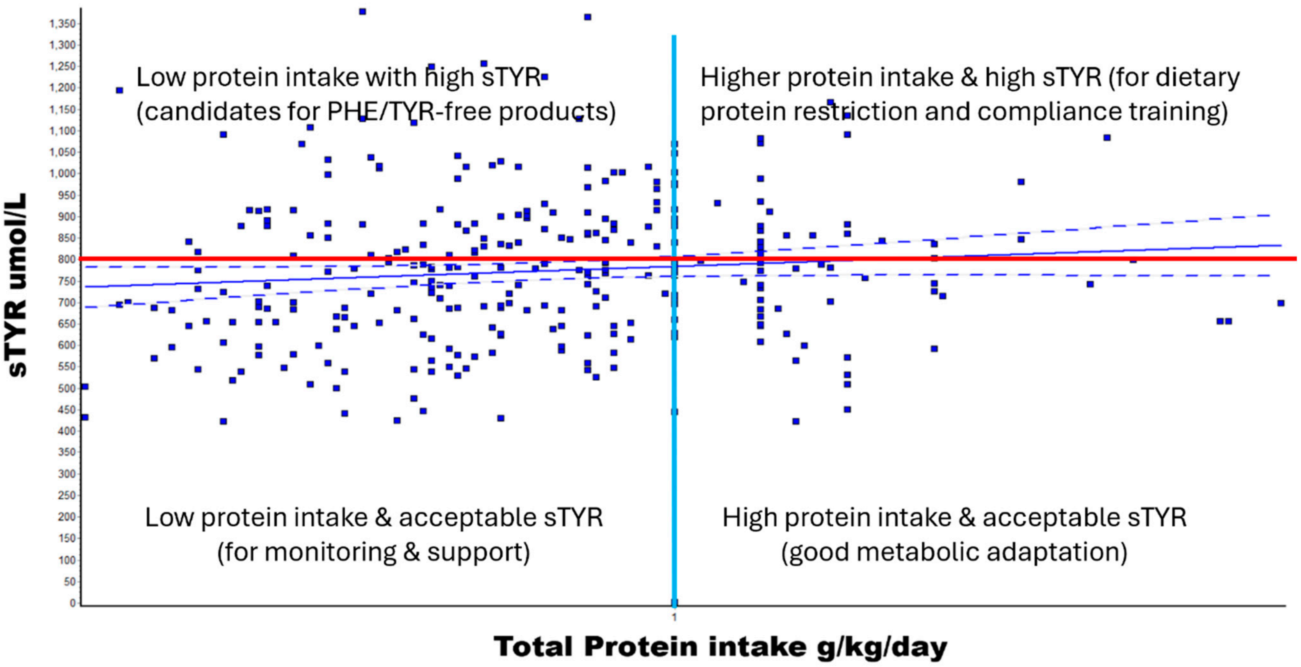

Supplement: Supplementary file 1 [file nutrients-16-02722-s001.zip › nutrients-3143629-supplementary.pdf]
